# Comparing the Potential of Marker-Assisted Selection and Genomic Prediction for Improving Rust Resistance in Hybrid Wheat

**DOI:** 10.3389/fpls.2020.594113

**Published:** 2020-10-28

**Authors:** Ulrike Beukert, Patrick Thorwarth, Yusheng Zhao, C. Friedrich H. Longin, Albrecht Serfling, Frank Ordon, Jochen C. Reif

**Affiliations:** ^1^Institute for Resistance Research and Stress Tolerance, Julius Kuehn-Institute (JKI) – Federal Research Centre for Cultivated Plants, Quedlinburg, Germany; ^2^State Plant Breeding Institute, University of Hohenheim, Stuttgart, Germany; ^3^Department of Breeding Research, Leibniz Institute of Plant Genetics and Crop Plant Research (IPK), Gatersleben, Germany

**Keywords:** resistance breeding, leaf rust (*Puccinia triticina*), stripe rust (*Puccinia striiformis* Westend), genome-wide selection, marker-assisted selection

## Abstract

Improving leaf rust and stripe rust resistance is a central goal in wheat breeding. The objectives of this study were to (1) elucidate the genetic basis of leaf rust and stripe rust resistance in a hybrid wheat population, (2) compare the findings using a previously published hybrid wheat data set, and (3) contrast the prediction accuracy with those of genome-wide prediction. The hybrid wheat population included 1,744 single crosses from 236 parental lines. The genotypes were fingerprinted using a 15k SNP array and evaluated for leaf rust and stripe rust resistance in multi-location field trials. We observed a high congruency of putative quantitative trait loci (QTL) for leaf rust resistance between both populations. This was not the case for stripe rust resistance. Accordingly, prediction accuracy of the detected QTL was moderate for leaf rust but low for stripe rust resistance. Genome-wide selection increased the prediction accuracy slightly for stripe rust albeit at a low level but not for leaf rust. Thus, our findings suggest that marker-assisted selection seems to be a robust and efficient tool to improve leaf rust resistance in European wheat hybrids.

## Introduction

Leaf rust caused by *Puccinia triticina* and stripe rust caused by *Puccinia striiformis* f. sp. *tritici* are important fungal diseases of wheat (Huerta-Espino et al., 2011). Both diseases can cause severe yield losses with simultaneous reduction of grain quality (Prescott et al., 1986; Chen, 2005). Breeding and growing of varieties carrying effective resistance genes against rust diseases is a sustainable solution to avoid or at least reduce yield and quality losses.

Molecular resistance breeding can simplify selection for rust resistances (Ordon et al., 1998; Miedaner and Korzun, 2012). Two complementary molecular breeding tools are marker-assisted selection (MAS) (Lande and Thompson, 1990) and genome-wide selection (GS) (Meuwissen et al., 2001). In MAS, the resistance of genotypes is estimated using a few diagnostic markers. MAS for rust resistance is often applied for pyramiding resistance genes within the same wheat cultivar (Singh et al., 2005). In GS, a complex genetic architecture is assumed and the resistance of genotypes is predicted using many markers (Meuwissen et al., 2001; Bernardo, 2008). GS is of particular interest to enrich the frequency of resistance alleles underlying quantitative disease resistance.

Currently, about 90 resistance genes for leaf rust (Lr-genes) and 80 resistance genes for stripe rust (Yr-genes) are known (McIntosh et al., 2017). Most of these genes are responsible for race-specific resistance (Singh et al., 2005; Bolton et al., 2008), which can easily be broken by rapidly evolving rust populations (Bolton et al., 2008; Serfling et al., 2013; Schwessinger, 2017). In contrast, only very few genes that quantitatively and stably reduce rust infestation are known to be non-race-specific such as *Lr34*/*Yr18* (Bolton et al., 2008; Chen, 2013) or *Yr29* (Chen, 2013). Resistance genes intensively utilized to enhance leaf rust resistance in European wheat cultivars are *Lr1*, *Lr3a*, *Lr10*, *Lr13*, *Lr14a*, *Lr17b*, *Lr20*, *Lr26*, and *Lr37* (Park et al., 2001; Pathan and Park, 2006), and those used to increase stripe rust resistance are *Yr1*, *Yr3, Yr4, Yr6*, *Yr7, Yr9*, *Yr17*, *Yr27*, *Yr32*, *YrHVII*, and *YrSP* (Pathan et al., 2008). Nevertheless, only *Lr1*, *Lr10*, *Lr21*, *Lr22a*, *Lr34*, *Yr10*, and *Yr15* have been cloned (Feuillet et al., 2003; Huang et al., 2003; Cloutier et al., 2007; Krattinger et al., 2011; Liu et al., 2014; Moore et al., 2015; Thind et al., 2017; Klymiuk et al., 2018) and diagnostic markers are available for *Lr1*, *Lr10*, *Lr26*, *Lr37*, *Yr4*, *Yr6*, *Yr7*, *Yr9*, *Yr17, Yr27*, and *Yr32* (Chen, 2005; Serfling et al., 2011; Zheng et al., 2017).

The potential of hybrid wheat breeding has been discussed in detail previously (e.g., Whitford et al., 2013). Hybrids are well-known to show a higher grain yield performance in combination with better yield stability compared to inbred lines. This fact supports the preference to expand the hybrid breeding strategy also into self-pollinating crops like wheat (Longin et al., 2013; Mühleisen et al., 2014; Jiang et al., 2017). Besides this main advantage, wheat hybrids are on average more resilient facing different biotic and abiotic stresses (Longin et al., 2013; Miedaner et al., 2013; Zhao et al., 2013; Gowda et al., 2014). The accumulation of various resistance genes within the same cultivar is important to keep existing resistances effective (Singh et al., 2005). An efficient way to stack a number of resistance genes is given by hybrid breeding (Longin et al., 2012), while its potential depends on the degree of dominance for special loci. Therefore, hybrid breeding might be a promising strategy to promote resistance breeding. Before routine application, it is pivotal to estimate the accuracy of MAS and GS. The accuracy should be determined in the relevant populations and have to be validated. The latter can be implemented either via cross-validation (Hjorth, 1994) or by validation with independent genotypes and environments (e.g., Jiang et al., 2016). Only a limited number of studies validated the accuracy of MAS and GS of rust resistances in wheat, and they rely on cross-validation (Gowda et al., 2014; Rutkoski et al., 2014; Juliana et al., 2017). Due to the restricted number of environments, cross-validation was performed for independent genotypes but not independent environments (Gowda et al., 2014; Rutkoski et al., 2014; Juliana et al., 2017), which is not reflecting the scenario relevant for breeding. Validation using independent samples, i.e., other genotypes from the same gene pool but tested in other environments, is, to our knowledge, lacking.

Our study is based on a comprehensive hybrid wheat population including 1,744 hybrids derived from crossing of 196 female and 40 male lines using an incomplete factorial mating design. The parents and hybrids were evaluated in multi-environment field trials for leaf rust and stripe rust resistance and genotyped using a 15k single-nucleotide polymorphism (SNP) array. The objectives of this study were to (1) dissect the genetic basis by performing genome-wide association mapping and identify quantitative trait loci (QTL) underlying leaf rust and stripe rust resistances, (2) compare the QTL making use of a previously published (Beukert et al., 2020) independent and comprehensive hybrid wheat population including 1,750 wheat hybrids and their 230 parental lines, and (3) contrast the prediction accuracy of MAS with that of GS.

## Materials and Methods

### Plant Material

This study comprised 196 female and 40 male elite winter wheat lines, their 1,744 single-cross hybrids, and 11 checks. The parental lines represent a wide range of diversity used in wheat breeding in Central Europe and were grouped into male and female lines according to their pollination capability, plant height, and flowering time. The elite lines were provided by the following 14 wheat breeding companies: BASF Agricultural Solutions GmbH, Deutsche Saatveredelung AG, KWS LOCHOW GmbH, Limagrain GmbH, Pflanzenzucht Oberlimpurg, RAGT-Saaten GmbH, Saatzucht Bauer GmbH, Saatzucht Josef Breun GmbH & Co. KG, Saatzucht Streng-Engelen GmbH & Co. KG, Secobra Saatzucht GmbH, Strube Research GmbH & Co. KG, Syngenta Seeds GmbH, Nordsaat Saatzucht GmbH, and W. von Borries-Eckendorf GmbH & Co. KG. The hybrids were produced following an incomplete factorial mating design using chemical hybridization agents (for details, see Zhao et al., in review).

### Field Trials and Experimental Design

Hybrids and their parental lines were grown in a multi-location field trial within the year 2018 to monitor their leaf rust and stripe rust resistance. Phenotypic data were collected in five different German environments in unreplicated trials (Table 1). Correlation of single locations was estimated based on overlapping check varieties using their raw data corrected for different design effects of the trials. The field locations of this study were correlated among each other with mean values of 0.44 and 0.39 for leaf rust and stripe rust resistance, respectively. In contrast to that, field trial locations mentioned in Beukert et al. (2020) showed mean correlations among each other of 0.29 and 0.70 for leaf rust and stripe rust resistance.

**TABLE 1:** Characterization of environments, in which leaf and stripe rust resistance were evaluated.

Location	Latitude	Longitude	Altitude (m)	Mean annual precipitation (mm)	Mean annual temperature (°C)
Hadmersleben	51.5837	11.1751	91	334	11.2
Gatersleben	51.824177	11.275706	112	510	9.8
Rosenthal	52.181889	10.105288	72	637	9.5
Seligenstadt	49.511630	10.06230	280	606	9.2
Feldkirchen	48.29217	48.29217	431	800	7.7

The genotypes were arranged into plots with a size of 1.00–2.70 m^2^ and randomized following an α lattice design considering environments as replications. The trials also included 11 check varieties (*JB Asano*, *Julius*, *RGT Reform*, *Colonia*, *KWS Loft*, *Rumor*, *Tobak*, *Elixer*, *Hybred*, *Hystar*, and *LG Alpha*) that were grown in each environment. Infection of genotypes occurred naturally, while local appearing rust populations consist of mixtures of different races showing similar composition in the observed German regions as well as similar virulence patterns across the examined years (2016–2018). Leaf rust and stripe rust infection was scored at the date of flowering (EC stage 65) on the flag leaf. An ordinal scale from 1 to 9 on the basis of the Bundessortenamt (2000) was used in order to score leaf rust as well as stripe rust infections, where 1 stands for minimal symptoms and 9 indicates extensive disease symptoms.

### Analysis of Phenotypic Data

Detection of outliers and estimation of variance components were conducted implementing the following mixed linear model:

$$y_{\mathrm{ijkl}}=\mu +e_{l}+b_{k|l}+g_{\mathrm{ij}}+m_{i}+f_{j}+s_{\mathrm{ij}}+{\left(\mathrm{me}\right)}_{\mathrm{il}}+{\left(\mathrm{fe}\right)}_{\mathrm{jl}}+\varepsilon_{\mathrm{ijkl}}$$

where *y*_ijkl_ is the performance of lines (*i=j*) or hybrids (*i*≠*j*) arising from a cross between the *i*th parent with the *j*th parent in the *k*th incomplete block in the *l*th environment. μ refers to the overall population mean. *e*_*l*_ is the effect of the *l*th environment, i.e., location by year combination; *b*_k—l_ represents the block effect of the *k*th block nested within the *l*th environment. *g*_ij_ was only modeled for the parental lines and stands for their genotypic effect. *m*_*i*_ and *f*_*j*_ were modeled for hybrids and are the GCA effects of the *i*th and *j*th of the male and female parent, respectively, *s*_*ij*_ symbolizes the SCA effect of the cross between the *i*th and *j*th parents. (*me*)_*il*_ as well as (*fe*)_*jl*_, which were only modeled for hybrids, are the interaction between the GCA effect of the *i*th and *j*th parent with the *l*th environment. ε_*ijkl*_ refers to the corresponding residuals. All effects except the intercept were modeled as random effects.

A second model was used to obtain best linear unbiased estimations (BLUEs) across environments:

$$y_{\mathrm{ikl}}=\mu +g_{i}+e_{l}+b_{k|l}+\varepsilon_{\mathrm{ikl}}$$

where *y*_*ikl*_ is the phenotypic observation of the *i*th genotype in the *k*th block at the *l*th environment. μ is the intercept, *g*_*i*_ symbolizes the genotypic effect of the *i*th individual, and *e*_*l*_ stands for the effect of the *l*th environment. *b*_*k—l*_ represents the block effect of the *k*th block nested within the *l*th environment, while ε_*ikl*_ is the residual error associated with the observation *y*_*ikl*_. The genotype effect was assumed as fixed to estimate the BLUEs, while all remaining effects were treated as random. Because of the unreplicated field trials, the residual effect within both equations was confounded with the SCA × environment interaction. Broad-sense heritability was calculated using variance component estimates of the first model as:

$$h^{2}=\frac{\sigma_{\mathrm{Genotype}}^{2}}{\sigma_{\mathrm{Phenotype}}^{2}}=\frac{\sigma_{\mathrm{Genotype}}^{2}}{\sigma_{\mathrm{Genotype}}^{2}+\frac{\sigma_{G\times E}^{2}}{N\,o.o\,f\,e\,n\,v\,i\,r\,o\,n\,m\,e\,n\,t}+\frac{\sigma_{\mathrm{error}}^{2}}{N\,o.o\,f\,e\,n\,v\,i\,r\,o\,n\,m\,e\,n\,t}}$$

Variance of genotypes was estimated as the sum of variance components of GCA and SCA effects. Variance of interaction effects of genotypes and environments was estimated as the sum of variance of GCA-by-environment interaction effects. Heritability for single locations was estimated to evaluate the quality of field trials. This was done by estimating the prediction abilities applying fivefold cross-validation as outlined in detail elsewhere (Schulthess et al., 2018).

### Genotypic Data Analysis

The extraction of DNA was conducted in compliance with known standard procedures (Stein et al., 2001). Parental lines were genotyped using a 15k SNP array containing a subset of the wheat 90k Illumina Infinium array (Wang et al., 2014). Composition of the 15k SNP chip and the genotyping was implemented by TraitGenetics GmbH.^1^ Population structure of the parental pools was examined by using the marker data to calculate Rogers’ distances and perform a principal component analysis (PCoA). Genotypic information was imputed in accordance to He et al. (2015). Quality filtering was performed and monomorphic markers, markers with missing values >5%, heterozygosity of >5% in inbred material, or a minor allele frequency (MAF) <5% were excluded. After this selection, 9,960 markers and 1,974 genotypes of high quality were left and used for association mapping. The procedure for the association mapping was previously described in detail by Liu et al. (2016). Briefly, we applied the following model:

Y=μ+Aa+Dd+Zz+ε

where *Y* describes BLUEs across the locations, μ is the vector of intercept effects, *a* symbolizes the vector of additive effects, *d* is a vector of dominance effects, *z* represents the vector of polygene background effects, and ε stands for the vector of residual effects. *A*, *D*, and *Z* were incidence matrices, which relates the BLUEs to the vectors *a*, *d*, and *z*. Further, a Bonferroni-corrected threshold of *P* <0.05 was applied to control for multiple testing. All statistical analyses were done using the software R (R Development Core Team, 2014) and the package ASReml-R 3.0 (Gilmour et al., 2009). The MAF for significantly associated markers was calculated and the linkage disequilibrium (LD) was assessed by the LD measure *r*^2^ (Weir, 1996) with an additional check for collinearity.

### Genomic Prediction of the Hybrid Performance

For genomic prediction, we implemented genomic best linear unbiased prediction (GBLUP), modeling both additive and dominance effects as:

$$Y=1_{n}\,\mu +g_{a}+g_{d}+e$$

*Y* refers to the genotype BLUEs of lines and hybrids; vector 1_*n*_ includes only ones and its element number is equal to the number of genotypes (*n*) used in this study; μ refers to the overall mean and was treated as fixed effect. The genotypic value was decomposed into an additive effect *g*_*a*_ and a dominance effect *g*_*d*_. The vector *e* represents the residual effect. More details on the implemented GBLUP model were described by Zhao et al. (2015). The prediction ability of leaf rust and stripe rust resistance was evaluated using a cross-validation scenario, which divides the total population into training and test population. Since relatedness strongly influences prediction accuracy (Habier et al., 2007), a cross-validation strategy was used considering three test sets with varying degrees of relatedness to the training population. Test set T2 was most closely related to the training population and included only hybrids derived from the same parents as the hybrids that had been evaluated. The less related test set T1 included hybrids sharing one parent with the hybrids in the training population. The least related test set T0 included only hybrids having no parents in common with the training population. Prediction ability was calculated as Pearson’s correlation coefficient between the observed and the predicted hybrid performance of test sets T2 to T0 including ∼750–30 hybrids, respectively.

### Validation of the Accuracy of GS vs. MAS Using an Independent Sample

A published hybrid wheat population, which was previously examined for their leaf rust and stripe rust resistance using a genome-wide association study by Beukert et al. (2020), was used in addition to the genotypes of this study to validate the accuracy of GS and MAS based on predictions following the GBLUP model. This previous published hybrid population included 1,750 wheat hybrids and their 230 parental lines, which were examined with the same 15k SNP array. Genotypes of this study were used as training population to train the statistical algorithm and in a further scenario as test population to prove its accuracy and vice versa, while the two examined sets show no overlapping genotypes. Only significantly associated markers identified in the association study were used to predict hybrid performance by MAS, while all available marker information was used performing GS. Marker effects were estimated based on the training population and applied to predict the performance of hybrids in the test population. Both prediction strategies were compared by observing their prediction ability based on the Pearson’s correlation coefficient between the observed and the predicted hybrid performance in the test population.

## Results

### Phenotypic Data of High Quality Were Generated in Comprehensive Field Trials

The hybrids and their parental lines were evaluated for leaf rust and stripe rust resistance in five environments, and BLUEs were estimated (Supplementary Table 1). The data quality was examined based on the prediction ability for single locations and ranged from 0.46 to 0.71 for leaf rust resistance, as well as from 0.24 to 0.60 for stripe rust resistance. The estimated heritability for leaf rust resistance for parents was *h*^2^ = 0.86 and that for hybrids was *h*^2^ = 0.84. Leaf rust resistance of the parental lines ranged from 1 to 8 with a mean of 3.97 (Table 2). The hybrid population covered a range from 1 to 8 with a mean value of 3.66. The assessment of different genotypes for stripe rust resistance resulted in heritability estimates for parents of *h*^2^ = 0.82 and for hybrids of *h*^2^ = 0.67. The parental pool showed a wide phenotypic distribution from 1 to 8 with an average of 2.51. In comparison, hybrids were less susceptible taking rating scores ranging from 1 to 6 with a mean value of 2.20.

**TABLE 2:** First- and second-degree statistics of 1,744 hybrids and their 236 parental lines observing leaf rust and stripe rust resistance.

	Leaf rust	Stripe rust
**Lines**		
*min*	0.85	0.73
*mean*	3.97	2.51
*max*	7.92	7.62
$\sigma_{G}^{2}$	2.22	1.74
*error*	0.69	0.79
*h*^2^	0.87	0.82
**Hybrids**		
*min*	0.45	0.56
*mean*	3.66	2.20
*max*	7.70	6.13
$\sigma_{G\,C\,A-f\,e\,m\,a\,l\,e}^{2}$	1.43	0.41
$\sigma_{G\,C\,A-m\,a\,l\,e}^{2}$	0.25	0.21
$\sigma_{\text{SCA}}^{2}$	0.19	0.18
$\sigma_{G\,C\,A\,f\,e\,m\,a\,l\,e\,x\,E\,n\,v}^{2}$	0.25	0.10
$\sigma_{G\,C\,A\,m\,a\,l\,e\,x\,E\,n\,v}^{2}$	0.07	0.04
*h*^2^	0.84	0.67
*error*	0.69	0.79
*genomich*^2^	0.46–0.71	0.24–0.60

*The parameters considered the phenotypic distribution, genotypic variance ($\sigma_{G}^{2}$), and heritability (*h*^2^), as well as the variance due general ($\sigma_{G\,C\,A}^{2}$) and special combining ability effects ($\sigma_{S\,C\,A}^{2}$).*

### Absence of Major Population Structure Among the Parental Lines

**FIGURE 1:**
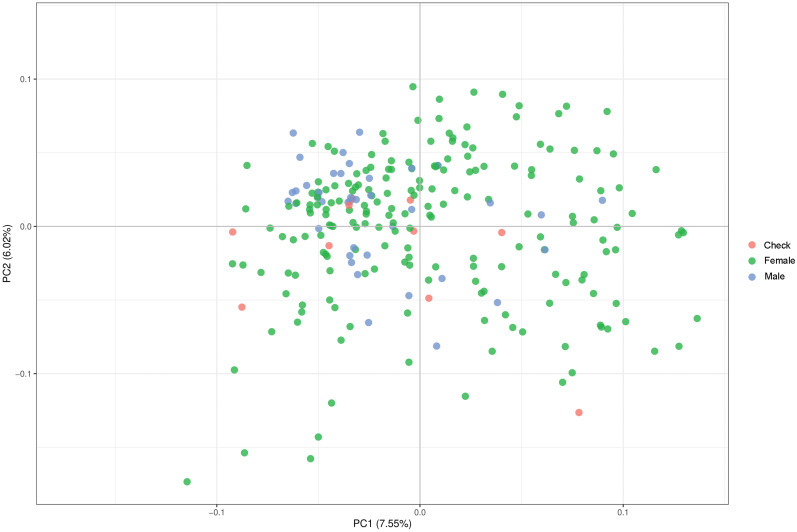
Result of principal component analysis (PCoA) observing the relationship and aggregation of parental lines and check varieties based on Rogers’ distances calculated using genome-wide marker data.

Parental lines were genotyped with genome-wide distributed SNP markers. We examined the population structure and relatedness of the 236 parental lines and implemented a PCoA (Figure 1) based on calculated Rogers’ distances. This procedure indicated that the population of parental lines is not structured and distinct parental pools are missing. Therefore, we corrected in the genome-wide association mapping for relatedness using a kinship matrix. For the validation of hybrid prediction methods, an additional population was observed showing no population structure within. A moderate relationship between the two different populations occurred due to the overlap of four male parents and a small effective population (Ne = 25) size of European elite lines used as parental pools for both hybrid populations (Supplementary Figure 1).

### GWAS Detected Several Putative QTL for Leaf Rust and Stripe Rust Resistance

**FIGURE 2:**
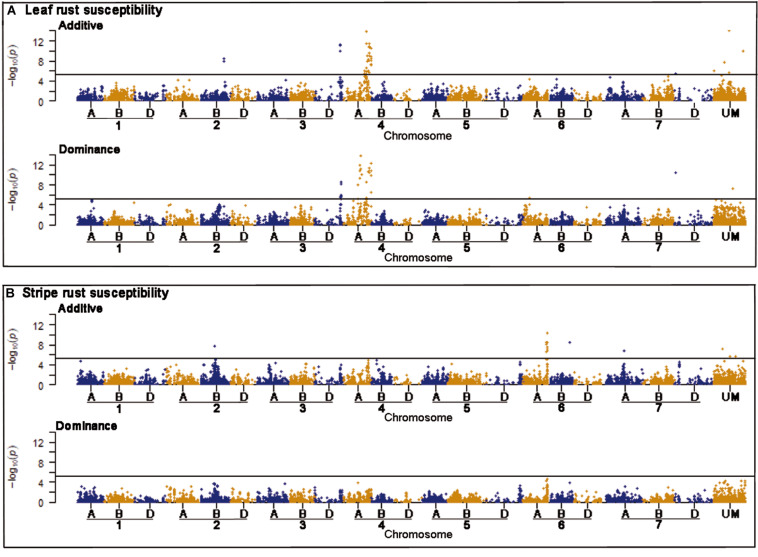
Manhattan plots of the genome-wide association scans for additive and dominance effects on leaf rust **(A)** and stripe rust resistance **(B)**. The horizontal line symbolizes the significant threshold of *P* < 0.05 applying Bonferroni correction. The hexaploid genome of bread wheat consists of 42 chromosomes combining the complete chromosomal sets of three different wild grasses, which are differentiated by the letters A, B, and D. The *x*-axis shows chromosomal location of the corresponding markers, while UM are unmapped markers.

Genome-wide association mapping scans were performed with a significance threshold of *P* < 0.05 applying Bonferroni correction for multiple testing. A total of 77 putative QTL were detected for leaf rust resistance. The putative QTL showed significant additive and dominance effects at similar frequencies and explained together 50.92% of the phenotypic variance (Supplementary Table 2). Strong associations for leaf rust resistance were found on chromosomes 3D and 4A (Figure 2). The most significant putative QTL underlying leaf rust resistance were identified on chromosome 4A with significant additive and dominance effects (Supplementary Table 2). There were 28 putative QTL on chromosome 4A in a region from 628 to 742 Mbp of the wheat reference genome (International Wheat Genome Sequencing Consortium [IWGSC], 2018). Four of the putative QTL on chromosome 4A and three putative QTL on 3D were identified within previously known genome regions that influence disease resistance (Table 3; International Wheat Genome Sequencing Consortium [IWGSC], 2018).

**TABLE 3:** Comparison of detected markers with a significant effect on leaf rust and stripe rust resistance with the location of previously known resistance genes within the reference genome.

Marker	Disease	Type	Chr.	Marker Pos.	Gene function	Physical gene Pos.	
	
(ID of reference gene)						Start (bp)	End (bp)
RAC875_c31922_138	Leaf rust	Add	3D	603,414,487	Disease resistance protein RPM1	603,414,478	603,419,584
(TraesCS3D01G522000)							
Kukri_c43464_89	Leaf rust	Add	3D	603,414,586	Disease resistance protein RPM1	603,414,478	603,419,584
(TraesCS3D01G522000)							
Kukri_c23354_183	Leaf rust	Add	3D	604,368,095	Leucin-rich repeat containing protein	604,367,900	604,371,312
(TraesCS3D01G524400)							
wsnp_Ex_c4331_7808746	Leaf rust	Add	4A	707,043,051	Protein enhanced disease resistance	707,040,590	707,048,030
(TraesCS4A01G437200)							
Excalibur_rep_c112888_602	Leaf rust	Add	4A	714,176,917	Disease resistance protein family (TIR-NBS-LRR class)	714,176,254	714,180,521
(TraesCS4A01G446700)							
RAC875_rep_c69632_65	Leaf rust	Add	4A	714,179,096	Disease resistance protein family (TIR-NBS-LRR class)	714,176,254	714,180,521
(TraesCS4A01G446700)							
BobWhite_c47168_289	Leaf rust	Add	4A	726,215,250	NBS-LRR disease resistance protein	726,212,910	726,217,457
(TraesCS4A01G461700)							
RAC875_c1226_652	Stripe rust	Add	2B	157,693,584	NBS-LRR disease resistance protein	157,688,966	157,696,282
(TraesCS2B01G182800)							

For stripe rust resistance, we detected 15 putative QTL all exclusively with significant additive effects (Figure 2). The 15 QTL explained together 19.53% of the phenotypic variance (Supplementary Table 3). The majority of seven putative QTL belonging to chromosome 6A and are physically located in the genomic region between 462 and 610 Mbp of the wheat reference genome (International Wheat Genome Sequencing Consortium [IWGSC], 2018). On chromosome 2B, we found a putative QTL for *RAC875_c1226_652*, which was located in a region of already known NBS-LRR genes (Table 3). Most of the putative QTL showed desired effects on stripe rust resistance (Supplementary Table 3).

### Prediction Ability of MAS Was Moderate for Leaf Rust but Low for Stripe Rust Resistance

We validated the potential of MAS using an independent, already published hybrid wheat data set (Beukert et al., 2020). The published data set included 1,750 wheat hybrids and their 230 parental lines, which were examined with the same 15k SNP array. Forty-five and six markers were identified to have a significant association to leaf rust and stripe rust resistance in both hybrid populations, respectively (Supplementary Table 4). The genotypes of one population were used for QTL detection and estimation of their effects based on a GBLUP approach, while the other population was taken as test population and vice versa. MAS was performed using only significantly associated markers detected in the GWAS to calculate the genomic values, while GS uses all available markers. The prediction ability of MAS, determined as the correlation between predicted and observed values, ranged from −0.07 for stripe rust resistance to 0.57 for leaf rust resistance (Table 4). The substantially higher prediction ability observed for leaf rust compared to stripe rust resistance was consistent and did not depend on which data set was used as training or test population. The low prediction ability for stripe rust resistance encouraged us to investigate genome-wide prediction as a possible tool to increase the prediction ability.

**TABLE 4:** Prediction ability implementing marker-assisted in comparison to genome-wide selection on different trainings and test populations to predict leaf rust and stripe rust resistance based on all available marker information in contrast to significant marker data out of association mapping.

	Leaf rust	Stripe rust
**Test population A**		
Marker-assisted selection	0.50	−0.07
Genome-wide prediction	0.43	0.21
**Test population B**		
Marker-assisted selection	0.57	0.19
Genome-wide prediction	0.50	0.16

*Within the first scenario, hybrid population A examined in detail by a previous study (Beukert et al., 2020) was set as test population, while genotypes introduced in this study representing population B were used as training population. Within a second scenario, the function of populations was changed vice versa.*

### MAS for Leaf Rust Resistance Exceeded GS

We implemented GBLUP considering additive and dominance effects. Interestingly, MAS outperformed GBLUP by 15% for leaf rust resistance. For stripe rust resistance, GBLUP improved the prediction ability, but only slightly up to a maximum value of 0.21 (Table 4). We additionally implemented a chessboard-like cross-validation within the data set of our study, thus confirming the low prediction ability between unrelated training and test population for stripe rust resistance (Supplementary Figure 2).

## Discussion

### Overlapping Check Varieties Indicated a Higher Pathogen Dynamic Underlying Leaf Rust Than Stripe Rust Infections

The results of Beukert et al. (2020) and our study are based on field trials that were possibly influenced by differences in the severity and composition of infections with *P. triticina* and *P. striiformis* f. sp. *tritici*. As an important quality control, we estimated the genomic heritability for leaf rust and stripe rust resistance, which allowed us to assess the disease pressure for each individual environment. The observed variation in genomic heritability on the one hand showed differences in disease pressure, but on the other hand, it reached a level that allowed a genetic differentiation. This differentiation resulted in heritability estimates for lines in the analysis over environments being above 0.8 for both traits.

**FIGURE 3:**
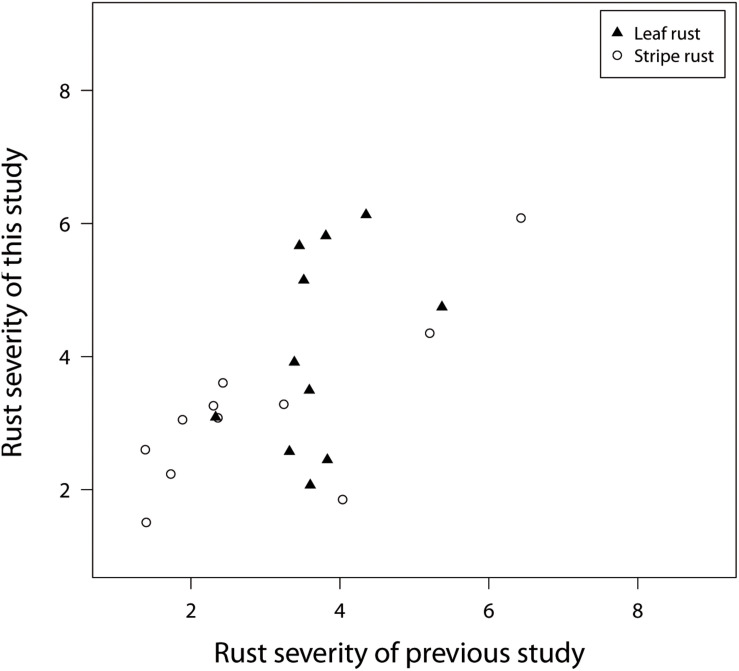
BLUEs representing leaf rust and stripe rust resistance of 11 check varieties, used in field trials of a previous study realized during field seasons in 2016 and 2017 in comparison to the field trial of 2018 belonging to this study.

Another interesting quality control is possible because Beukert et al. (2020) and the current study used 11 overlapping check genotypes, which were relevant released varieties. The checks showed a limited phenotypic diversity with regard to rust resistance and were at least moderately resistant to stripe rust and leaf rust (Figure 3). In addition, a more precise characterization of the pathogenic variation across field locations was not possible due to the lack of knowledge about resistance genes fixed in each check variety. Despite these shortcomings, the comparison of disease resistance for the overlapping check varieties showed correlations of *r* = 0.37 for leaf rust and *r* = 0.77 for stripe rust resistance (Figure 3). These findings suggest an excellent agreement for stripe rust disease screening and points to inconsistencies for leaf rust and to a climatic-dependent dynamic in the pathogen population of *P. triticina*. Such a dynamic in the pathogen population can of course also influence the concordance of further downstream analyses.

### MAS for Leaf Rust Resistance Is a Promising Molecular Breeding Strategy

**FIGURE 4:**
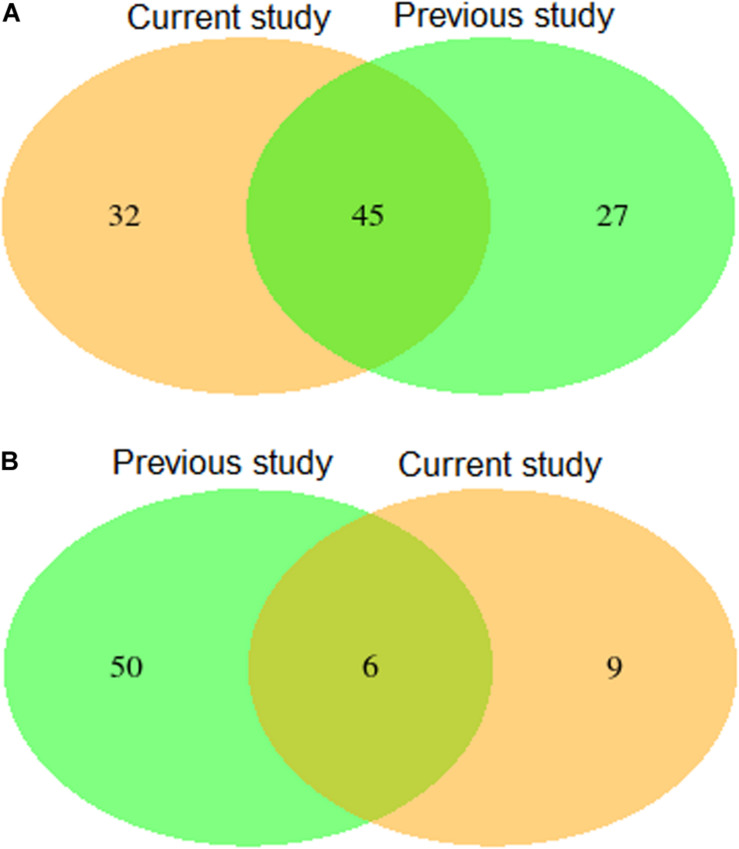
Venn diagram showing numbers of detected and overlapping QTLs for leaf rust **(A)** and stripe rust resistance **(B)** comparing results of the present study with a previous published study by Beukert et al. (2020) (previous study).

Validation using independent data sets revealed a substantial overlap of 45 putative QTL (Figure 4 and Supplementary Table 5) for leaf rust resistance, as found in our study and in Beukert et al. (2020). The 45 putative SNPs cover regions on chromosome 3D, 4A, and 7D that most likely harbor relevant genes contributing to leaf rust resistance. In particular, the putative QTL on chromosome 4A were consistently the most significant in both studies. A more detailed exome association analysis (Liu et al., 2019) based on the population underlying the published study by Beukert et al. (2020) suggests that the underlying resistance gene is *Lr34-B*, a homolog of the cloned gene *Lr34* (Krattinger et al., 2011).

In hybrid wheat breeding, the degree of dominance of the QTL determines the optimal breeding strategy (for a detailed discussion, see Beukert et al., 2020). Therefore, we compared the degree of dominance for the overlapping 45 putative QTL and observed that all of them showed the desired negative degree of dominance in both data sets (Supplementary Tables 2, 4). This suggests that these putative QTL are interesting targets to develop resistance strategies for hybrid wheat breeding. The nice overlap of the putative QTL for leaf rust resistance is also reflected in validated prediction abilities of MAS amounting to 0.50 and 0.57 (Table 4). In comparison, Juliana et al. (2017) reported a lower prediction ability of MAS for leaf rust resistance of 0.21 based on germplasm of CIMMYT’s spring wheat breeding programs fingerprinted using genotyping by sequencing. In summary, our results underline the possibility of a robust and efficient prediction of leaf rust resistance in European germplasm based on a limited number of diagnostic markers.

We also performed GS in the hope of further increasing the prediction ability for leaf rust resistance. Nevertheless, we could not boost the prediction ability of MAS (0.50 and 0.57) and observed values of 0.43 and 0.50 in the validation of genome-wide prediction based on independent data sets (Table 4). These values outperformed the prediction ability of 0.34 reported by Juliana et al. (2017) for leaf rust resistance in an inbred line population. Therefore, these results indicate that MAS is the method of choice to support the breeding of resistance to leaf rust. Nevertheless, further studies are needed to validate these findings.

The implementation of MAS promotes the breeding efficiency (Miedaner and Korzun, 2012; Bassi et al., 2015) and is especially interesting to accumulate favorable genes within early plant generations (Bonnett et al., 2005): for instance, MAS was successfully performed in commercial wheat breeding to fix the rust resistance genes *Lr34* and *Yr36* (Miedaner and Korzun, 2012). A challenge when increasing the number of diagnostic markers for multi-trait selection is of course undesired linkage, which requires large population sizes. Nevertheless, MAS is of high relevance for the early stages of single seed descent (SSD)-based programs allowing a negative selection for rust resistance in combination with an excellent balance between costs and informative data (Gupta et al., 2010).

### Need to Further Increase the Prediction Ability for Stripe Rust Resistance

Validation using independent data sets revealed only a minor overlap of six putative QTL (Figure 4 and Supplementary Table 5) for stripe rust resistance, as found in our study and in Beukert et al. (2020). An example for the lack of consistency is the strong peak on chromosome 2A detected by Beukert et al. (2020) that could not be confirmed in this study. The inconsistency also holds true for the degree of dominance. Only one of the six putative QTL showed a significant dominance effect causing a reduced susceptibility in both populations (Supplementary Tables 3, 4). Effects of all other common QTL resulted in differences between both studies. The minor overlap of the putative QTL for stripe rust resistance is also reflected in validated prediction abilities of MAS amounting to −0.07 and 0.19 (Table 4). In comparison, Juliana et al. (2017) reported higher predicting abilities of MAS for stripe rust resistance of, on average, 0.32 using CIMMYT’s spring wheat breeding populations fingerprinted with genotyping by sequencing. The low prediction ability observed in our study can be caused among other factors by the presence of many minor genes resulting in a complex genetic architecture.

We therefore also performed GS, which is more suitable to predict the performance of complex traits. The validated prediction ability changed only marginally compared to those of MAS (Table 4), which is in contrast to the results of Juliana et al. (2017) reporting average prediction abilities of 0.35. These findings suggest that the success of genome-wide prediction strongly depends on the underlying germplasm base and the used marker system and shows the need for innovations to boost prediction ability of stripe rust resistance in European wheat lines.

## Conclusion

Within this study, we applied a genome-wide association study to investigate the genetic architecture of leaf rust and stripe rust resistance in a European hybrid wheat population. In particular, identified loci significantly associated to leaf rust resistance were comparable with another hybrid population based on the same genetic origin. Afterward, this previous published hybrid population was used in addition to perform MAS in contrast to GS to compare their prediction accuracy. Applying MAS resulted in moderate and low prediction abilities for leaf rust and stripe rust resistance, respectively. GS led to slightly increased prediction abilities for stripe rust in contrast to leaf rust resistance. Accordingly, MAS seems to be a viable option to improve the level of leaf rust resistance in European wheat. In contrast, the general validity of our results observing stripe rust resistance is limited by the very low prediction accuracy. Previous studies of Ornella et al. (2012) and Juliana et al. (2019) validated genomic prediction for rust resistance using independent samples from CIMMYT’s breeding material. Therefore, it is already known that prediction accuracy is highly influenced by germplasm base, population structure, marker system, pathogenic system, and phenotyping conditions. Within this study, the prediction ability for stripe rust was in the range of values reported for grain yield, taking into account the situation of unrelated training and test populations (Zhao et al., 2015). The considerable difference in prediction abilities observed between leaf rust and stripe rust are surprising, given the fact that the selection gain for stripe rust resistance in Central Europe has been high in recent decades, indicating the presence of major genes (Hovmøller, 2007; Pathan et al., 2008; Sørensen et al., 2014). Together with the discrepancies compared to previous studies investigating the prediction abilities of stripe rust resistance in spring wheat (Juliana et al., 2017), the need for further research is evident. Beside this, the application of a genome-wide prediction model treating the major QTLs as fixed effects (Rutkoski et al., 2014) would be an attractive aspect in order to study the resilience of different prediction strategies. A further interesting option is the use of other genotyping platforms such as RenSeq (Jupe et al., 2013; Steuernagel et al., 2016) or whole genome sequencing (Huang et al., 2009; Brenchley et al., 2012; Chapman et al., 2015), which hold the promise to detect the relevant QTL of stripe rust resistance and thereby boost the prediction ability.

## Data Availability Statement

The datasets generated for this study are available in the Supplementary Material.

## Author Contributions

JR and FL designed the study. PT and YZ curated phenotypic and genomic data. PT and UB performed the analyses. UB and JR wrote the manuscript with input from all co-authors. All authors contributed to the article and approved the submitted version.

## Conflict of Interest

The authors declare that the research was conducted in the absence of any commercial or financial relationships that could be construed as a potential conflict of interest.

## Footnotes

1 http://www.traitgenetics.com
