# Supplementary material for: Comparing the Potential of Marker-Assisted Selection and Genomic Prediction for Improving Rust Resistance in Hybrid Wheat
Source: Front Plant Sci. 2020 Oct 28;11:594113. doi: 10.3389/fpls.2020.594113 (PMC7655876; doi:10.3389/fpls.2020.594113)
Supplement: Supplementary file 1 [file Data_Sheet_1.docx]

Supplementary Material

# Supplementary Figures and Tables

## Supplementary Figures


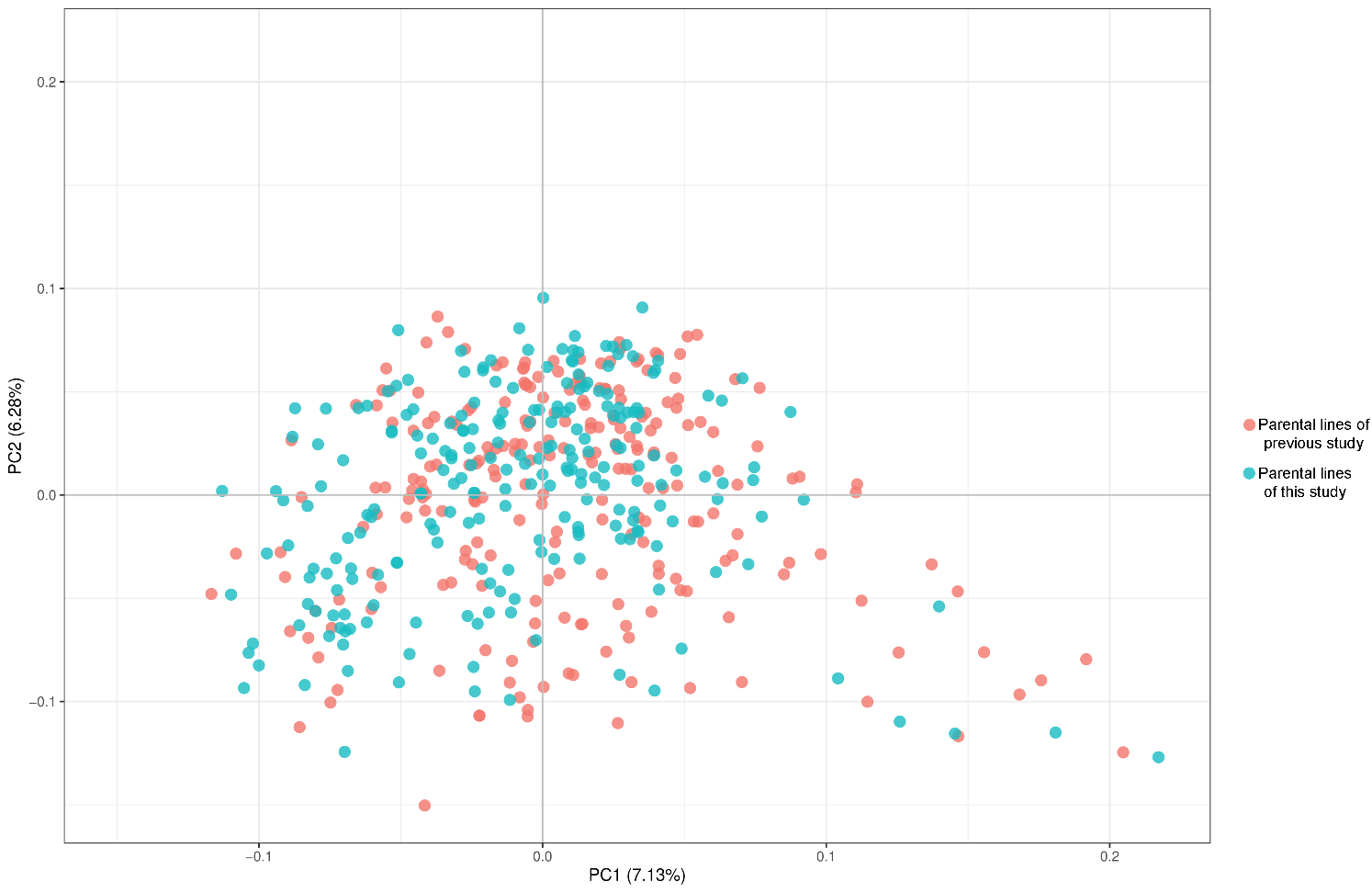


**Suppl. Figure 1:** Result of principal component analysis (PCA) based on Rogers` distances observing the relationship between hybrid population examined in this study and a previous described population (Beukert et al. 2020) basing both on European wheat elite material.


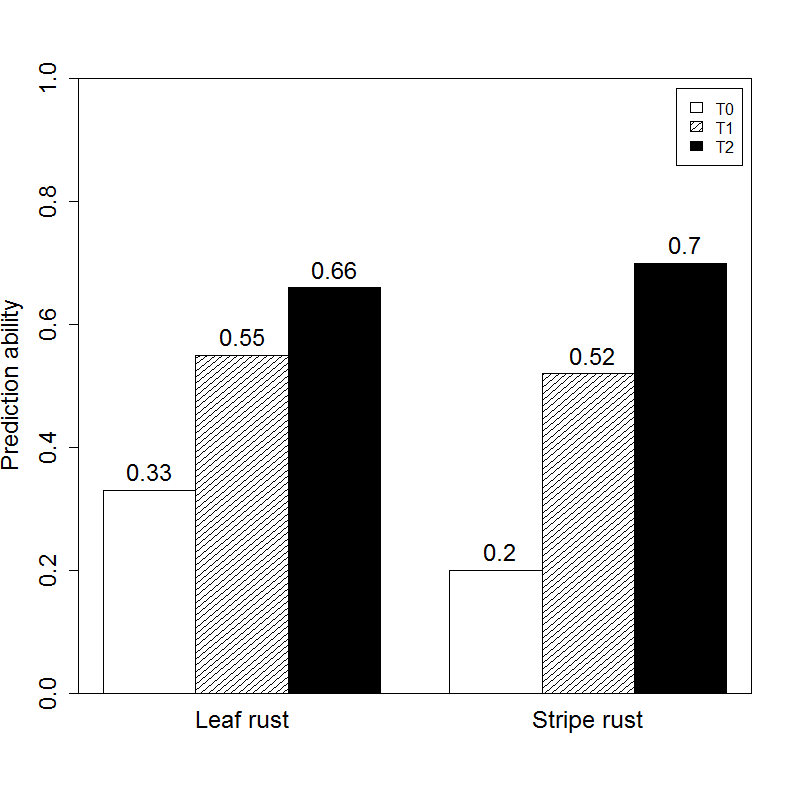


**Suppl. Figure 2:** Ability of predicting leaf rust and stripe rust resistance by performing genome-wide prediction under consideration of different relation scenarios between trainings and test population. Hybrids included in test set T2 have both parents in common with hybrids of training population, while T0 represents completely different parents between test set and training population.

## Supplementary Tables

**Suppl. Table 1:** Estimated BLUEs observing leaf rust and stripe rust resistance of check varieties, tested hybrids, and parental lines. Values based on phenotyping scores of the flag leaf at the date of flowering (EC stage 65) regarding multi-locations. The used ordinal phenotyping scale ranges from 1 to 9 on the basis of the Bundessortenamt (2000), where one symbolizes minimal symptoms and nine indicates extensive disease symptoms.

| **Genotype** | **Leaf rust** | **Stripe rust** |
| --- | --- | --- |
| Colonia | 3.92 | 2.60 |
| Elixer | 3.49 | 2.23 |
| Hybred | 6.13 | 3.08 |
| Hystar | 2.45 | 1.85 |
| JBAsano | 5.81 | 6.08 |
| Julius | 5.15 | 3.26 |
| KWSLoft | 2.07 | 4.35 |
| LGAlpha | 2.57 | 3.60 |
| RGTReform | 3.09 | 3.05 |
| Rumor | 5.67 | 3.28 |
| Tobak | 4.74 | 1.51 |
| Female301 | 4.32 | 3.18 |
| Female302 | 6.11 | 3.41 |
| Female304 | 5.53 | 2.01 |
| Female305 | 6.13 | 3.06 |
| Female306 | 2.77 | 2.41 |
| Female307 | 6.90 | 2.37 |
| Female308 | 4.25 | 3.71 |
| Female309 | 1.96 | 2.09 |
| Female310 | 4.48 | 2.27 |
| Female311 | 5.24 | 2.32 |
| Female312 | 5.11 | 3.72 |
| Female313 | 3.09 | 2.99 |
| Female314 | 1.93 | 1.98 |
| Female315 | 1.44 | 1.71 |
| Female316 | 3.99 | 2.22 |
| Female317 | 4.17 | 1.90 |
| Female318 | 3.29 | 1.95 |
| Female319 | 6.21 | 2.49 |
| Female320 | 5.95 | 1.37 |
| Female321 | 5.70 | 2.14 |
| Female322 | 1.90 | 2.03 |
| Female323 | 2.09 | 1.47 |
| Female324 | 1.98 | 1.70 |
| Female325 | 2.01 | 2.34 |
| Female326 | 5.53 | 4.06 |
| Female327 | 3.38 | 3.62 |
| Female328 | 4.26 | 5.83 |
| Female329 | 1.95 | 3.27 |
| Female330 | 2.50 | 2.55 |
| Female331 | 3.90 | 1.54 |
| Female332 | 6.56 | 4.11 |
| Female333 | 4.91 | 3.06 |
| Female334 | 4.74 | 2.87 |
| Female335 | 2.02 | 1.85 |
| Female336 | 5.27 | 3.29 |
| Female337 | 6.43 | 2.32 |
| Female338 | 6.02 | 1.70 |
| Female339 | 4.40 | 5.40 |
| Female341 | 5.13 | 1.51 |
| Female342 | 6.53 | 2.27 |
| Female343 | 6.45 | 1.59 |
| Female344 | 2.94 | 1.60 |
| Female345 | 5.15 | 1.96 |
| Female346 | 4.87 | 2.06 |
| Female347 | 5.20 | 3.96 |
| Female348 | 5.00 | 1.66 |
| Female349 | 3.68 | 2.41 |
| Female350 | 1.39 | 3.05 |
| Female351 | 1.41 | 2.36 |
| Female352 | 5.82 | 1.70 |
| Female353 | 2.92 | 3.44 |
| Female355 | 4.31 | 1.40 |
| Female356 | 1.23 | 2.53 |
| Female357 | 4.04 | 1.69 |
| Female358 | 4.46 | 1.53 |
| Female359 | 5.05 | 1.89 |
| Female360 | 5.46 | 3.28 |
| Female361 | 4.93 | 2.79 |
| Female362 | 2.56 | 1.94 |
| Female363 | 2.65 | 1.69 |
| Female364 | 1.58 | 1.98 |
| Female365 | 4.12 | 1.50 |
| Female366 | 1.35 | 1.83 |
| Female367 | 2.07 | 2.47 |
| Female368 | 1.57 | 2.31 |
| Female369 | 4.67 | 1.86 |
| Female370 | 2.88 | 1.78 |
| Female371 | 3.38 | 1.80 |
| Female372 | 1.62 | 4.52 |
| Female373 | 3.72 | 3.88 |
| Female374 | 6.68 | 5.58 |
| Female375 | 3.22 | 4.48 |
| Female376 | 2.18 | 2.51 |
| Female377 | 3.98 | 1.80 |
| Female378 | 6.24 | 2.11 |
| Female379 | 4.20 | 2.76 |
| Female380 | 4.55 | 2.45 |
| Female381 | 5.20 | 1.61 |
| Female382 | 5.21 | 1.43 |
| Female383 | 4.38 | 3.80 |
| Female384 | 3.92 | 1.41 |
| Female385 | 2.73 | 1.47 |
| Female386 | 1.90 | 4.46 |
| Female387 | 1.45 | 1.32 |
| Female388 | 1.98 | 4.84 |
| Female389 | 0.90 | 2.96 |
| Female390 | 4.26 | 1.34 |
| Female391 | 7.27 | 4.03 |
| Female392 | 5.71 | 1.06 |
| Female393 | 1.79 | 4.61 |
| Female394 | 6.36 | 1.70 |
| Female395 | 7.92 | 2.46 |
| Female396 | 5.51 | 1.74 |
| Female397 | 3.96 | 2.64 |
| Female398 | 1.33 | 1.25 |
| Female399 | 4.28 | 2.03 |
| Female400 | 3.23 | 3.49 |
| Female401 | 1.09 | 1.31 |
| Female402 | 2.37 | 3.23 |
| Female403 | 1.71 | 1.97 |
| Female404 | 4.91 | 1.46 |
| Female405 | 5.81 | 2.71 |
| Female406 | 3.74 | 2.03 |
| Female407 | 2.35 | 4.71 |
| Female408 | 3.53 | 2.47 |
| Female409 | 6.11 | 1.43 |
| Female410 | 5.30 | 2.51 |
| Female411 | 5.25 | 1.54 |
| Female412 | 4.17 | 2.48 |
| Female413 | 6.00 | 1.69 |
| Female414 | 5.08 | 2.74 |
| Female415 | 2.58 | 6.78 |
| Female416 | 2.22 | 2.32 |
| Female417 | 4.71 | 3.71 |
| Female418 | 5.60 | 2.28 |
| Female419 | 2.96 | 1.92 |
| Female420 | 1.81 | 2.57 |
| Female421 | 5.36 | 2.72 |
| Female422 | 1.65 | 2.10 |
| Female423 | 5.84 | 1.52 |
| Female424 | 4.32 | 4.30 |
| Female425 | 3.45 | 2.02 |
| Female426 | 5.74 | 3.27 |
| Female427 | 4.49 | 1.38 |
| Female428 | 5.18 | 1.68 |
| Female429 | 3.91 | 1.35 |
| Female430 | 4.20 | 2.05 |
| Female431 | 3.13 | 1.79 |
| Female432 | 5.66 | 1.57 |
| Female433 | 2.96 | 2.61 |
| Female434 | 4.45 | 1.81 |
| Female435 | 5.31 | 1.64 |
| Female436 | 4.79 | 2.35 |
| Female437 | 6.84 | 5.70 |
| Female438 | 5.88 | 2.22 |
| Female439 | 4.39 | 2.15 |
| Female440 | 3.02 | 4.23 |
| Female441 | 2.71 | 3.46 |
| Female442 | 5.93 | 3.03 |
| Female443 | 4.84 | 1.66 |
| Female444 | 3.25 | 1.60 |
| Female445 | 3.88 | 2.52 |
| Female446 | 6.20 | 2.62 |
| Female447 | 3.30 | 3.61 |
| Female448 | 4.76 | 2.02 |
| Female449 | 2.72 | 1.42 |
| Female450 | 5.99 | 1.42 |
| Female451 | 5.45 | 1.89 |
| Female453 | 3.78 | 1.90 |
| Female454 | 2.11 | 2.62 |
| Female455 | 4.11 | 1.90 |
| Female456 | 6.37 | 2.82 |
| Female457 | 2.32 | 5.46 |
| Female458 | 1.89 | 1.34 |
| Female459 | 2.89 | 1.24 |
| Female460 | 2.80 | 1.44 |
| Female461 | 4.52 | 2.15 |
| Female462 | 2.95 | 2.02 |
| Female463 | 4.73 | 2.05 |
| Female464 | 5.20 | 2.28 |
| Female465 | 1.28 | 2.52 |
| Female466 | 3.47 | 4.35 |
| Female467 | 5.60 | 3.13 |
| Female468 | 1.80 | 1.88 |
| Female469 | 4.23 | 1.36 |
| Female470 | 1.75 | 3.29 |
| Female471 | 3.29 | 1.67 |
| Female472 | 3.06 | 3.40 |
| Female473 | 2.55 | 4.75 |
| Female474 | 2.66 | 1.58 |
| Female475 | 1.28 | 2.77 |
| Female476 | 1.76 | 2.00 |
| Female477 | 0.85 | 1.33 |
| Female478 | 3.16 | 2.12 |
| Female479 | 2.07 | 1.24 |
| Female480 | 1.77 | 1.77 |
| Female481 | 6.41 | 3.64 |
| Female482 | 3.50 | 4.57 |
| Female483 | 2.82 | 1.29 |
| Female484 | 6.55 | 1.76 |
| Female485 | 5.31 | 5.12 |
| Female486 | 4.34 | 5.43 |
| Female487 | 6.71 | 7.06 |
| Female488 | 6.26 | 4.64 |
| Female489 | 6.11 | 2.40 |
| Female490 | 5.39 | 2.51 |
| Female491 | 4.64 | 3.56 |
| Female492 | 6.15 | 2.49 |
| Female493 | 4.90 | 5.57 |
| Female494 | 1.88 | 2.33 |
| Female495 | 2.62 | 7.83 |
| Female496 | 3.76 | 3.11 |
| Female497 | 2.66 | 2.03 |
| Female498 | 2.91 | 2.26 |
| Female499 | 5.01 | 1.38 |
| Female500 | 3.39 | 1.41 |
| Male001 | 5.42 | 1.60 |
| Male004 | 2.80 | 3.31 |
| Male005 | 3.13 | 1.41 |
| Male009 | 3.36 | 1.92 |
| Male301 | 5.05 | 1.49 |
| Male302 | 2.87 | 1.81 |
| Male303 | 4.43 | 2.62 |
| Male304 | 2.99 | 1.35 |
| Male309 | 5.92 | 2.69 |
| Male310 | 2.20 | 1.25 |
| Male311 | 2.12 | 2.52 |
| Male312 | 3.55 | 3.44 |
| Male313 | 4.23 | 1.80 |
| Male314 | 3.95 | 1.87 |
| Male315 | 2.84 | 1.93 |
| Male316 | 3.45 | 2.07 |
| Male317 | 4.81 | 3.50 |
| Male318 | 3.71 | 1.42 |
| Male319 | 3.55 | 1.73 |
| Male320 | 3.31 | 2.34 |
| Male321 | 6.47 | 1.82 |
| Male322 | 2.80 | 1.38 |
| Male323 | 5.71 | 2.91 |
| Male324 | 3.54 | 1.90 |
| Male325 | 2.55 | 1.41 |
| Male326 | 3.01 | 2.02 |
| Male327 | 1.84 | 2.09 |
| Male328 | 3.37 | 2.05 |
| Male329 | 1.66 | 3.69 |
| Male330 | 5.93 | 2.45 |
| Male331 | 4.72 | 1.92 |
| Male332 | 4.76 | 1.57 |
| Male333 | 6.46 | 2.33 |
| Male334 | 3.96 | 2.64 |
| Male335 | 5.01 | 1.87 |
| Male336 | 5.65 | 1.85 |
| Male337 | 6.08 | 3.15 |
| Male338 | 3.22 | 3.36 |
| Male339 | 2.42 | 1.55 |
| Male340 | 3.92 | 5.53 |
| Male001 x Female301 | 4.20 | 2.56 |
| Male001 x Female305 | 6.07 | 2.44 |
| Male001 x Female313 | 5.19 | 3.42 |
| Male001 x Female316 | 4.67 | 2.37 |
| Male001 x Female325 | 1.90 | 3.24 |
| Male001 x Female327 | 2.98 | 2.55 |
| Male001 x Female331 | 2.89 | 1.67 |
| Male001 x Female333 | 4.21 | 2.15 |
| Male001 x Female335 | 2.38 | 1.43 |
| Male001 x Female338 | 4.92 | 1.50 |
| Male001 x Female343 | 4.96 | 1.77 |
| Male001 x Female347 | 3.68 | 2.58 |
| Male001 x Female348 | 5.14 | 1.63 |
| Male001 x Female355 | 4.57 | 2.11 |
| Male001 x Female356 | 4.50 | 1.91 |
| Male001 x Female359 | 4.07 | 1.93 |
| Male001 x Female361 | 5.02 | 2.39 |
| Male001 x Female367 | 1.89 | 1.53 |
| Male001 x Female377 | 4.58 | 1.86 |
| Male001 x Female384 | 3.43 | 1.37 |
| Male001 x Female389 | 1.60 | 2.46 |
| Male001 x Female391 | 7.03 | 2.70 |
| Male001 x Female392 | 5.71 | 1.47 |
| Male001 x Female394 | 5.26 | 1.67 |
| Male001 x Female396 | 5.01 | 1.42 |
| Male001 x Female397 | 3.62 | 1.66 |
| Male001 x Female398 | 2.26 | 1.39 |
| Male001 x Female399 | 3.74 | 1.35 |
| Male001 x Female400 | 4.77 | 2.56 |
| Male001 x Female402 | 4.64 | 1.49 |
| Male001 x Female403 | 0.92 | 1.88 |
| Male001 x Female404 | 6.00 | 1.55 |
| Male001 x Female406 | 4.52 | 2.01 |
| Male001 x Female407 | 2.50 | 4.62 |
| Male001 x Female408 | 4.96 | 1.99 |
| Male001 x Female409 | 6.11 | 1.87 |
| Male001 x Female410 | 5.06 | 1.62 |
| Male001 x Female411 | 4.49 | 1.87 |
| Male001 x Female413 | 5.36 | 1.51 |
| Male001 x Female414 | 3.77 | 1.82 |
| Male001 x Female415 | 3.38 | 4.40 |
| Male001 x Female416 | 2.12 | 2.00 |
| Male001 x Female417 | 5.27 | 2.32 |
| Male001 x Female426 | 5.17 | 2.69 |
| Male001 x Female427 | 5.38 | 1.75 |
| Male001 x Female428 | 4.83 | 1.42 |
| Male001 x Female430 | 4.61 | 2.14 |
| Male001 x Female432 | 6.32 | 1.89 |
| Male001 x Female436 | 4.66 | 1.94 |
| Male001 x Female442 | 4.84 | 2.25 |
| Male001 x Female443 | 5.45 | 1.97 |
| Male001 x Female444 | 4.95 | 1.70 |
| Male001 x Female445 | 5.66 | 1.85 |
| Male001 x Female450 | 4.82 | 1.87 |
| Male001 x Female457 | 2.85 | 3.58 |
| Male001 x Female459 | 1.51 | 1.57 |
| Male001 x Female465 | 2.56 | 1.27 |
| Male001 x Female466 | 3.33 | 2.28 |
| Male001 x Female470 | 2.48 | 2.29 |
| Male001 x Female471 | 2.01 | 1.81 |
| Male001 x Female473 | 1.72 | 2.09 |
| Male001 x Female477 | 1.96 | 1.45 |
| Male001 x Female482 | 3.20 | 3.53 |
| Male001 x Female484 | 5.19 | 1.72 |
| Male001 x Female490 | 5.21 | 1.67 |
| Male001 x Female496 | 4.49 | 2.13 |
| Male001 x Female499 | 3.67 | 1.89 |
| Male004 x Female302 | 3.91 | 3.49 |
| Male004 x Female304 | 5.20 | 3.23 |
| Male004 x Female307 | 5.23 | 2.09 |
| Male004 x Female309 | 1.37 | 1.87 |
| Male004 x Female314 | 2.45 | 1.59 |
| Male004 x Female317 | 4.77 | 3.22 |
| Male004 x Female322 | 2.16 | 2.22 |
| Male004 x Female329 | 1.94 | 2.96 |
| Male004 x Female332 | 4.51 | 4.10 |
| Male004 x Female334 | 4.22 | 3.27 |
| Male004 x Female338 | 6.48 | 1.87 |
| Male004 x Female341 | 4.52 | 1.54 |
| Male004 x Female343 | 5.30 | 1.76 |
| Male004 x Female345 | 5.87 | 3.65 |
| Male004 x Female349 | 5.50 | 2.00 |
| Male004 x Female355 | 3.76 | 1.59 |
| Male004 x Female363 | 3.28 | 1.84 |
| Male004 x Female364 | 1.47 | 2.35 |
| Male004 x Female366 | 1.61 | 3.33 |
| Male004 x Female373 | 1.87 | 5.01 |
| Male004 x Female381 | 5.85 | 2.06 |
| Male004 x Female385 | 2.21 | 1.39 |
| Male004 x Female387 | 2.34 | 1.46 |
| Male004 x Female395 | 5.90 | 2.10 |
| Male004 x Female398 | 2.20 | 2.47 |
| Male004 x Female400 | 4.61 | 4.42 |
| Male004 x Female401 | 4.77 | 1.70 |
| Male004 x Female403 | 0.97 | 1.31 |
| Male004 x Female404 | 5.80 | 2.47 |
| Male004 x Female418 | 5.74 | 3.93 |
| Male004 x Female423 | 3.94 | 2.19 |
| Male004 x Female425 | 2.96 | 2.32 |
| Male004 x Female433 | 2.23 | 3.31 |
| Male004 x Female435 | 3.23 | 2.76 |
| Male004 x Female440 | 3.23 | 3.71 |
| Male004 x Female450 | 3.73 | 1.44 |
| Male004 x Female451 | 5.18 | 3.74 |
| Male004 x Female458 | 2.48 | 1.65 |
| Male004 x Female462 | 3.40 | 2.21 |
| Male004 x Female472 | 3.45 | 4.63 |
| Male004 x Female474 | 2.97 | 3.62 |
| Male004 x Female475 | 1.31 | 4.13 |
| Male004 x Female476 | 1.23 | 2.13 |
| Male004 x Female480 | 1.48 | 1.45 |
| Male004 x Female481 | 6.30 | 3.49 |
| Male004 x Female484 | 5.43 | 3.24 |
| Male004 x Female488 | 4.35 | 4.77 |
| Male004 x Female489 | 4.98 | 1.82 |
| Male004 x Female492 | 5.36 | 3.13 |
| Male004 x Female494 | 2.97 | 2.84 |
| Male005 x Female344 | 3.65 | 1.34 |
| Male005 x Female352 | 5.57 | 2.10 |
| Male005 x Female353 | 2.53 | 2.51 |
| Male005 x Female358 | 4.34 | 1.62 |
| Male005 x Female359 | 4.44 | 1.69 |
| Male005 x Female367 | 1.54 | 2.45 |
| Male005 x Female380 | 3.95 | 1.32 |
| Male005 x Female405 | 2.23 | 1.57 |
| Male005 x Female410 | 4.50 | 1.85 |
| Male005 x Female419 | 3.13 | 1.98 |
| Male005 x Female448 | 3.03 | 1.35 |
| Male005 x Female449 | 4.10 | 1.77 |
| Male005 x Female497 | 3.30 | 2.00 |
| Male009 x Female302 | 5.05 | 3.09 |
| Male009 x Female308 | 4.26 | 3.17 |
| Male009 x Female310 | 4.19 | 3.18 |
| Male009 x Female311 | 6.00 | 2.21 |
| Male009 x Female318 | 3.51 | 1.33 |
| Male009 x Female319 | 4.52 | 2.03 |
| Male009 x Female324 | 2.60 | 1.28 |
| Male009 x Female326 | 5.56 | 3.10 |
| Male009 x Female330 | 3.71 | 1.30 |
| Male009 x Female337 | 5.64 | 2.37 |
| Male009 x Female346 | 3.10 | 2.14 |
| Male009 x Female355 | 4.04 | 1.72 |
| Male009 x Female360 | 5.17 | 1.38 |
| Male009 x Female362 | 3.58 | 2.18 |
| Male009 x Female365 | 4.82 | 1.53 |
| Male009 x Female369 | 4.88 | 1.75 |
| Male009 x Female371 | 3.23 | 2.83 |
| Male009 x Female375 | 4.20 | 2.94 |
| Male009 x Female379 | 5.44 | 2.50 |
| Male009 x Female382 | 4.84 | 1.68 |
| Male009 x Female383 | 3.46 | 3.73 |
| Male009 x Female386 | 2.09 | 4.65 |
| Male009 x Female393 | 2.46 | 3.21 |
| Male009 x Female402 | 4.03 | 2.47 |
| Male009 x Female409 | 5.01 | 1.99 |
| Male009 x Female414 | 3.12 | 2.90 |
| Male009 x Female421 | 5.52 | 2.51 |
| Male009 x Female422 | 2.33 | 1.73 |
| Male009 x Female424 | 5.21 | 4.39 |
| Male009 x Female431 | 3.32 | 1.56 |
| Male009 x Female437 | 5.15 | 4.45 |
| Male009 x Female438 | 5.62 | 1.95 |
| Male009 x Female441 | 2.32 | 3.64 |
| Male009 x Female447 | 2.72 | 3.70 |
| Male009 x Female449 | 3.81 | 2.11 |
| Male009 x Female453 | 2.70 | 1.36 |
| Male009 x Female454 | 2.04 | 3.37 |
| Male009 x Female455 | 4.52 | 2.22 |
| Male009 x Female460 | 1.56 | 1.65 |
| Male009 x Female462 | 3.22 | 2.82 |
| Male009 x Female468 | 2.15 | 1.72 |
| Male009 x Female475 | 3.00 | 3.02 |
| Male009 x Female479 | 1.39 | 1.30 |
| Male009 x Female491 | 2.82 | 3.39 |
| Male301 x Female301 | 4.77 | 1.55 |
| Male301 x Female305 | 5.10 | 3.12 |
| Male301 x Female313 | 5.24 | 2.12 |
| Male301 x Female316 | 6.75 | 1.73 |
| Male301 x Female325 | 3.35 | 1.75 |
| Male301 x Female327 | 2.85 | 2.34 |
| Male301 x Female331 | 2.52 | 2.01 |
| Male301 x Female333 | 4.00 | 2.17 |
| Male301 x Female335 | 2.49 | 1.65 |
| Male301 x Female343 | 5.74 | 2.27 |
| Male301 x Female347 | 5.65 | 2.39 |
| Male301 x Female361 | 5.39 | 1.51 |
| Male301 x Female384 | 4.23 | 1.22 |
| Male301 x Female389 | 1.35 | 1.57 |
| Male301 x Female391 | 7.70 | 2.34 |
| Male301 x Female392 | 5.31 | 2.08 |
| Male301 x Female396 | 4.84 | 2.06 |
| Male301 x Female397 | 5.32 | 1.26 |
| Male301 x Female399 | 4.12 | 1.59 |
| Male301 x Female400 | 5.89 | 1.71 |
| Male301 x Female402 | 6.78 | 1.85 |
| Male301 x Female407 | 3.60 | 1.71 |
| Male301 x Female413 | 4.94 | 1.73 |
| Male301 x Female415 | 2.94 | 1.69 |
| Male301 x Female427 | 5.65 | 1.65 |
| Male301 x Female430 | 3.08 | 2.18 |
| Male301 x Female432 | 5.14 | 2.06 |
| Male301 x Female436 | 4.16 | 1.84 |
| Male301 x Female443 | 6.22 | 1.95 |
| Male301 x Female445 | 6.13 | 2.36 |
| Male301 x Female450 | 5.20 | 1.39 |
| Male301 x Female457 | 2.38 | 1.60 |
| Male301 x Female459 | 2.10 | 1.37 |
| Male301 x Female466 | 2.99 | 1.93 |
| Male301 x Female470 | 2.06 | 1.62 |
| Male301 x Female471 | 2.59 | 1.42 |
| Male301 x Female473 | 1.70 | 1.85 |
| Male301 x Female477 | 3.12 | 1.37 |
| Male301 x Female484 | 5.69 | 1.34 |
| Male301 x Female490 | 5.19 | 1.96 |
| Male301 x Female499 | 3.78 | 1.44 |
| Male302 x Female307 | 5.47 | 2.68 |
| Male302 x Female309 | 2.74 | 1.54 |
| Male302 x Female317 | 5.31 | 2.82 |
| Male302 x Female322 | 2.08 | 2.25 |
| Male302 x Female329 | 3.31 | 1.96 |
| Male302 x Female334 | 4.24 | 1.82 |
| Male302 x Female338 | 5.64 | 1.87 |
| Male302 x Female363 | 2.21 | 1.50 |
| Male302 x Female364 | 1.34 | 2.45 |
| Male302 x Female368 | 1.19 | 1.58 |
| Male302 x Female372 | 2.52 | 2.73 |
| Male302 x Female373 | 1.85 | 2.45 |
| Male302 x Female376 | 3.65 | 2.30 |
| Male302 x Female398 | 1.94 | 1.60 |
| Male302 x Female400 | 4.13 | 2.82 |
| Male302 x Female401 | 4.28 | 2.35 |
| Male302 x Female403 | 1.48 | 1.33 |
| Male302 x Female425 | 3.55 | 1.98 |
| Male302 x Female440 | 3.63 | 1.95 |
| Male302 x Female451 | 5.34 | 1.64 |
| Male302 x Female458 | 2.55 | 1.53 |
| Male302 x Female464 | 6.01 | 2.51 |
| Male302 x Female472 | 2.64 | 2.66 |
| Male302 x Female474 | 2.79 | 1.47 |
| Male302 x Female480 | 2.53 | 1.83 |
| Male302 x Female481 | 6.44 | 2.31 |
| Male302 x Female484 | 5.55 | 1.86 |
| Male302 x Female489 | 4.85 | 1.50 |
| Male302 x Female492 | 5.41 | 1.98 |
| Male303 x Female302 | 5.97 | 3.56 |
| Male303 x Female308 | 3.62 | 3.29 |
| Male303 x Female310 | 4.38 | 3.03 |
| Male303 x Female311 | 5.43 | 2.51 |
| Male303 x Female318 | 3.37 | 1.84 |
| Male303 x Female324 | 3.03 | 1.47 |
| Male303 x Female326 | 5.56 | 4.12 |
| Male303 x Female330 | 4.15 | 2.66 |
| Male303 x Female349 | 4.42 | 2.88 |
| Male303 x Female350 | 1.96 | 1.94 |
| Male303 x Female355 | 4.27 | 1.58 |
| Male303 x Female360 | 4.97 | 2.67 |
| Male303 x Female362 | 3.77 | 1.67 |
| Male303 x Female371 | 4.65 | 2.94 |
| Male303 x Female375 | 5.54 | 4.29 |
| Male303 x Female378 | 5.35 | 2.22 |
| Male303 x Female382 | 4.12 | 3.76 |
| Male303 x Female383 | 3.61 | 3.82 |
| Male303 x Female393 | 1.97 | 4.63 |
| Male303 x Female397 | 4.38 | 2.63 |
| Male303 x Female399 | 4.66 | 1.58 |
| Male303 x Female402 | 3.12 | 3.15 |
| Male303 x Female409 | 5.51 | 1.72 |
| Male303 x Female414 | 5.02 | 2.20 |
| Male303 x Female420 | 1.30 | 2.32 |
| Male303 x Female421 | 5.19 | 3.31 |
| Male303 x Female422 | 2.81 | 3.23 |
| Male303 x Female424 | 4.75 | 3.07 |
| Male303 x Female431 | 3.86 | 2.11 |
| Male303 x Female437 | 6.21 | 4.89 |
| Male303 x Female438 | 6.27 | 2.01 |
| Male303 x Female441 | 3.31 | 3.92 |
| Male303 x Female447 | 3.42 | 3.93 |
| Male303 x Female449 | 3.92 | 3.46 |
| Male303 x Female453 | 1.99 | 1.44 |
| Male303 x Female454 | 2.43 | 2.72 |
| Male303 x Female455 | 4.23 | 2.72 |
| Male303 x Female460 | 2.30 | 3.14 |
| Male303 x Female462 | 4.00 | 3.73 |
| Male303 x Female468 | 1.80 | 2.69 |
| Male303 x Female475 | 1.74 | 4.38 |
| Male303 x Female476 | 2.14 | 2.03 |
| Male303 x Female486 | 5.18 | 4.21 |
| Male303 x Female491 | 4.28 | 3.80 |
| Male304 x Female306 | 2.43 | 1.56 |
| Male304 x Female311 | 2.57 | 2.25 |
| Male304 x Female312 | 2.92 | 5.07 |
| Male304 x Female313 | 2.39 | 3.28 |
| Male304 x Female315 | 2.32 | 1.56 |
| Male304 x Female321 | 1.37 | 2.55 |
| Male304 x Female323 | 1.22 | 1.86 |
| Male304 x Female328 | 2.17 | 3.40 |
| Male304 x Female333 | 3.25 | 3.94 |
| Male304 x Female336 | 2.28 | 2.43 |
| Male304 x Female339 | 3.02 | 2.50 |
| Male304 x Female342 | 3.20 | 1.83 |
| Male304 x Female344 | 2.02 | 1.94 |
| Male304 x Female351 | 1.19 | 2.00 |
| Male304 x Female352 | 3.03 | 1.54 |
| Male304 x Female353 | 2.52 | 1.55 |
| Male304 x Female358 | 3.24 | 1.41 |
| Male304 x Female359 | 2.12 | 1.54 |
| Male304 x Female360 | 2.75 | 2.31 |
| Male304 x Female367 | 1.30 | 2.09 |
| Male304 x Female370 | 2.78 | 1.44 |
| Male304 x Female374 | 3.03 | 2.52 |
| Male304 x Female380 | 1.58 | 1.52 |
| Male304 x Female388 | 1.43 | 3.85 |
| Male304 x Female389 | 1.42 | 2.56 |
| Male304 x Female390 | 3.66 | 1.11 |
| Male304 x Female396 | 4.14 | 1.47 |
| Male304 x Female397 | 3.02 | 1.45 |
| Male304 x Female398 | 2.36 | 1.61 |
| Male304 x Female409 | 3.14 | 1.94 |
| Male304 x Female410 | 2.27 | 2.28 |
| Male304 x Female412 | 1.80 | 2.19 |
| Male304 x Female414 | 2.45 | 1.47 |
| Male304 x Female415 | 2.33 | 6.25 |
| Male304 x Female419 | 2.61 | 1.53 |
| Male304 x Female425 | 3.67 | 2.69 |
| Male304 x Female434 | 2.47 | 1.54 |
| Male304 x Female439 | 2.06 | 2.73 |
| Male304 x Female446 | 4.12 | 3.27 |
| Male304 x Female448 | 2.81 | 1.39 |
| Male304 x Female449 | 1.62 | 3.43 |
| Male304 x Female456 | 2.29 | 1.38 |
| Male304 x Female457 | 1.95 | 3.01 |
| Male304 x Female463 | 2.85 | 2.10 |
| Male304 x Female467 | 3.35 | 1.92 |
| Male304 x Female469 | 3.26 | 2.13 |
| Male304 x Female478 | 2.36 | 1.56 |
| Male304 x Female483 | 1.87 | 1.71 |
| Male304 x Female485 | 2.97 | 4.43 |
| Male304 x Female487 | 3.03 | 2.56 |
| Male304 x Female493 | 2.53 | 1.80 |
| Male304 x Female495 | 1.84 | 4.30 |
| Male304 x Female497 | 1.99 | 3.01 |
| Male304 x Female498 | 2.49 | 1.74 |
| Male304 x Female499 | 4.57 | 1.33 |
| Male304 x Female500 | 3.08 | 1.76 |
| Male309 x Female305 | 5.35 | 4.28 |
| Male309 x Female314 | 1.49 | 1.57 |
| Male309 x Female316 | 5.89 | 3.47 |
| Male309 x Female320 | 5.99 | 2.91 |
| Male309 x Female323 | 1.52 | 3.04 |
| Male309 x Female325 | 3.65 | 3.39 |
| Male309 x Female327 | 2.41 | 4.64 |
| Male309 x Female332 | 5.13 | 3.40 |
| Male309 x Female334 | 5.04 | 3.69 |
| Male309 x Female336 | 4.01 | 3.83 |
| Male309 x Female338 | 5.51 | 2.20 |
| Male309 x Female347 | 6.04 | 3.47 |
| Male309 x Female349 | 4.34 | 3.42 |
| Male309 x Female351 | 2.28 | 5.08 |
| Male309 x Female355 | 3.90 | 2.36 |
| Male309 x Female357 | 6.07 | 2.63 |
| Male309 x Female358 | 4.74 | 1.35 |
| Male309 x Female360 | 6.50 | 3.47 |
| Male309 x Female367 | 1.15 | 3.76 |
| Male309 x Female383 | 3.27 | 3.62 |
| Male309 x Female386 | 1.69 | 4.85 |
| Male309 x Female388 | 1.36 | 5.65 |
| Male309 x Female392 | 5.18 | 1.97 |
| Male309 x Female396 | 4.59 | 1.82 |
| Male309 x Female402 | 3.43 | 2.98 |
| Male309 x Female407 | 2.64 | 4.37 |
| Male309 x Female415 | 2.95 | 4.27 |
| Male309 x Female417 | 4.92 | 3.67 |
| Male309 x Female419 | 2.40 | 2.08 |
| Male309 x Female421 | 5.41 | 3.47 |
| Male309 x Female424 | 3.32 | 4.35 |
| Male309 x Female429 | 4.45 | 2.77 |
| Male309 x Female432 | 4.15 | 1.59 |
| Male309 x Female436 | 5.23 | 2.69 |
| Male309 x Female446 | 5.36 | 2.56 |
| Male309 x Female448 | 2.95 | 2.07 |
| Male309 x Female451 | 4.97 | 3.10 |
| Male309 x Female456 | 5.38 | 1.92 |
| Male309 x Female459 | 0.90 | 1.59 |
| Male309 x Female461 | 4.45 | 3.14 |
| Male309 x Female463 | 3.85 | 3.02 |
| Male309 x Female467 | 5.96 | 2.86 |
| Male309 x Female470 | 1.70 | 3.81 |
| Male309 x Female473 | 1.58 | 3.66 |
| Male309 x Female480 | 1.53 | 1.40 |
| Male309 x Female482 | 2.77 | 3.95 |
| Male309 x Female489 | 4.67 | 2.79 |
| Male309 x Female494 | 3.01 | 2.70 |
| Male309 x Female498 | 3.11 | 3.29 |
| Male309 x Female500 | 3.80 | 3.09 |
| Male310 x Female302 | 6.41 | 3.09 |
| Male310 x Female306 | 3.29 | 3.32 |
| Male310 x Female315 | 3.58 | 3.56 |
| Male310 x Female317 | 5.25 | 2.20 |
| Male310 x Female322 | 5.97 | 3.23 |
| Male310 x Female324 | 3.13 | 2.72 |
| Male310 x Female330 | 4.65 | 1.78 |
| Male310 x Female335 | 2.69 | 1.62 |
| Male310 x Female337 | 5.90 | 1.47 |
| Male310 x Female344 | 4.52 | 2.16 |
| Male310 x Female348 | 4.77 | 2.12 |
| Male310 x Female350 | 1.24 | 2.34 |
| Male310 x Female353 | 2.64 | 2.93 |
| Male310 x Female363 | 2.79 | 2.31 |
| Male310 x Female370 | 2.70 | 1.44 |
| Male310 x Female377 | 6.09 | 3.08 |
| Male310 x Female384 | 3.75 | 1.49 |
| Male310 x Female387 | 3.84 | 1.49 |
| Male310 x Female389 | 1.34 | 3.35 |
| Male310 x Female395 | 5.15 | 1.56 |
| Male310 x Female397 | 4.51 | 2.49 |
| Male310 x Female401 | 3.23 | 1.77 |
| Male310 x Female404 | 5.76 | 2.19 |
| Male310 x Female414 | 3.96 | 2.59 |
| Male310 x Female416 | 3.45 | 2.88 |
| Male310 x Female418 | 5.47 | 3.11 |
| Male310 x Female420 | 2.20 | 1.78 |
| Male310 x Female422 | 3.68 | 1.29 |
| Male310 x Female425 | 2.69 | 2.02 |
| Male310 x Female431 | 4.08 | 2.88 |
| Male310 x Female434 | 5.80 | 1.88 |
| Male310 x Female445 | 5.87 | 2.36 |
| Male310 x Female447 | 4.74 | 3.04 |
| Male310 x Female450 | 5.12 | 1.58 |
| Male310 x Female454 | 2.69 | 2.78 |
| Male310 x Female457 | 2.20 | 3.96 |
| Male310 x Female460 | 2.56 | 2.67 |
| Male310 x Female462 | 4.61 | 4.59 |
| Male310 x Female466 | 2.84 | 2.78 |
| Male310 x Female468 | 1.55 | 2.07 |
| Male310 x Female472 | 3.32 | 3.88 |
| Male310 x Female477 | 2.23 | 1.52 |
| Male310 x Female481 | 6.55 | 3.34 |
| Male310 x Female483 | 2.23 | 2.08 |
| Male310 x Female493 | 5.80 | 4.56 |
| Male310 x Female499 | 4.00 | 1.46 |
| Male311 x Female302 | 3.71 | 3.86 |
| Male311 x Female306 | 2.49 | 2.68 |
| Male311 x Female315 | 2.84 | 3.42 |
| Male311 x Female317 | 4.29 | 3.17 |
| Male311 x Female322 | 2.57 | 4.31 |
| Male311 x Female324 | 1.72 | 3.35 |
| Male311 x Female326 | 2.95 | 4.47 |
| Male311 x Female330 | 2.91 | 2.27 |
| Male311 x Female335 | 1.77 | 1.37 |
| Male311 x Female337 | 2.99 | 1.87 |
| Male311 x Female344 | 1.49 | 2.99 |
| Male311 x Female348 | 2.18 | 2.07 |
| Male311 x Female350 | 1.27 | 2.51 |
| Male311 x Female363 | 1.56 | 2.51 |
| Male311 x Female370 | 1.56 | 1.38 |
| Male311 x Female377 | 3.04 | 3.44 |
| Male311 x Female384 | 1.58 | 1.59 |
| Male311 x Female387 | 1.97 | 1.53 |
| Male311 x Female389 | 1.35 | 3.82 |
| Male311 x Female395 | 2.93 | 1.39 |
| Male311 x Female397 | 2.16 | 2.72 |
| Male311 x Female401 | 2.12 | 2.70 |
| Male311 x Female404 | 3.91 | 2.15 |
| Male311 x Female414 | 2.61 | 3.00 |
| Male311 x Female416 | 1.31 | 3.48 |
| Male311 x Female418 | 3.05 | 4.03 |
| Male311 x Female420 | 1.60 | 3.08 |
| Male311 x Female422 | 2.09 | 3.65 |
| Male311 x Female425 | 2.80 | 4.34 |
| Male311 x Female431 | 2.03 | 3.24 |
| Male311 x Female434 | 2.20 | 1.88 |
| Male311 x Female445 | 4.18 | 2.84 |
| Male311 x Female450 | 2.06 | 1.60 |
| Male311 x Female454 | 1.20 | 3.79 |
| Male311 x Female457 | 2.57 | 4.45 |
| Male311 x Female460 | 1.70 | 3.53 |
| Male311 x Female462 | 1.49 | 4.28 |
| Male311 x Female466 | 1.52 | 4.76 |
| Male311 x Female468 | 1.59 | 2.67 |
| Male311 x Female472 | 2.31 | 2.70 |
| Male311 x Female477 | 1.43 | 1.86 |
| Male311 x Female479 | 1.64 | 1.79 |
| Male311 x Female481 | 2.39 | 3.52 |
| Male311 x Female483 | 1.83 | 2.23 |
| Male311 x Female493 | 2.51 | 4.77 |
| Male311 x Female497 | 1.72 | 2.26 |
| Male311 x Female499 | 1.85 | 2.24 |
| Male312 x Female305 | 5.21 | 2.30 |
| Male312 x Female314 | 2.86 | 1.47 |
| Male312 x Female316 | 5.67 | 2.26 |
| Male312 x Female323 | 2.39 | 1.56 |
| Male312 x Female325 | 2.44 | 1.60 |
| Male312 x Female327 | 2.69 | 2.25 |
| Male312 x Female332 | 4.79 | 1.63 |
| Male312 x Female334 | 4.47 | 1.64 |
| Male312 x Female336 | 3.16 | 1.82 |
| Male312 x Female338 | 4.75 | 1.41 |
| Male312 x Female347 | 4.40 | 2.13 |
| Male312 x Female349 | 5.04 | 1.32 |
| Male312 x Female351 | 1.30 | 1.78 |
| Male312 x Female355 | 3.43 | 1.95 |
| Male312 x Female357 | 3.78 | 1.73 |
| Male312 x Female358 | 4.51 | 1.62 |
| Male312 x Female360 | 5.27 | 2.15 |
| Male312 x Female367 | 1.14 | 2.23 |
| Male312 x Female383 | 3.64 | 2.14 |
| Male312 x Female386 | 1.62 | 3.04 |
| Male312 x Female388 | 1.49 | 2.73 |
| Male312 x Female392 | 3.72 | 1.55 |
| Male312 x Female396 | 3.93 | 1.72 |
| Male312 x Female402 | 3.68 | 2.46 |
| Male312 x Female407 | 3.30 | 2.31 |
| Male312 x Female415 | 4.02 | 2.25 |
| Male312 x Female417 | 4.28 | 1.83 |
| Male312 x Female419 | 1.64 | 1.38 |
| Male312 x Female421 | 3.72 | 2.29 |
| Male312 x Female424 | 4.26 | 2.73 |
| Male312 x Female429 | 3.47 | 1.29 |
| Male312 x Female432 | 5.18 | 1.35 |
| Male312 x Female436 | 2.74 | 1.12 |
| Male312 x Female446 | 3.85 | 1.53 |
| Male312 x Female448 | 2.68 | 1.27 |
| Male312 x Female451 | 3.56 | 1.43 |
| Male312 x Female456 | 2.66 | 1.67 |
| Male312 x Female459 | 1.84 | 1.44 |
| Male312 x Female461 | 3.35 | 1.33 |
| Male312 x Female463 | 3.12 | 1.61 |
| Male312 x Female467 | 3.78 | 1.75 |
| Male312 x Female470 | 2.81 | 1.50 |
| Male312 x Female473 | 1.62 | 1.30 |
| Male312 x Female480 | 1.71 | 1.98 |
| Male312 x Female482 | 2.94 | 1.93 |
| Male312 x Female489 | 3.79 | 1.58 |
| Male312 x Female494 | 1.26 | 1.92 |
| Male312 x Female498 | 2.45 | 1.72 |
| Male312 x Female500 | 3.03 | 1.78 |
| Male313 x Female302 | 6.18 | 3.12 |
| Male313 x Female306 | 4.30 | 1.71 |
| Male313 x Female315 | 3.47 | 2.19 |
| Male313 x Female317 | 5.63 | 2.43 |
| Male313 x Female322 | 5.00 | 3.12 |
| Male313 x Female324 | 1.49 | 1.91 |
| Male313 x Female326 | 5.10 | 3.67 |
| Male313 x Female330 | 4.66 | 2.21 |
| Male313 x Female335 | 1.69 | 1.58 |
| Male313 x Female337 | 5.86 | 2.05 |
| Male313 x Female344 | 4.32 | 2.30 |
| Male313 x Female348 | 4.89 | 1.69 |
| Male313 x Female363 | 2.37 | 1.43 |
| Male313 x Female370 | 1.92 | 1.89 |
| Male313 x Female384 | 2.58 | 1.45 |
| Male313 x Female389 | 2.06 | 2.93 |
| Male313 x Female395 | 4.56 | 1.53 |
| Male313 x Female397 | 3.59 | 2.40 |
| Male313 x Female404 | 4.95 | 1.92 |
| Male313 x Female414 | 4.61 | 2.58 |
| Male313 x Female418 | 5.46 | 1.78 |
| Male313 x Female420 | 1.15 | 1.43 |
| Male313 x Female422 | 2.12 | 1.87 |
| Male313 x Female425 | 2.80 | 2.43 |
| Male313 x Female431 | 3.68 | 2.23 |
| Male313 x Female434 | 4.40 | 1.88 |
| Male313 x Female445 | 5.79 | 2.13 |
| Male313 x Female450 | 2.51 | 2.06 |
| Male313 x Female460 | 1.94 | 1.39 |
| Male313 x Female466 | 2.50 | 2.02 |
| Male313 x Female472 | 2.82 | 2.34 |
| Male313 x Female477 | 2.20 | 1.99 |
| Male313 x Female481 | 6.74 | 2.71 |
| Male313 x Female483 | 2.30 | 1.59 |
| Male313 x Female499 | 2.90 | 1.89 |
| Male314 x Female305 | 6.70 | 3.17 |
| Male314 x Female316 | 4.52 | 1.20 |
| Male314 x Female320 | 4.41 | 2.16 |
| Male314 x Female323 | 1.88 | 1.62 |
| Male314 x Female325 | 2.34 | 2.48 |
| Male314 x Female327 | 2.31 | 3.23 |
| Male314 x Female332 | 5.43 | 2.24 |
| Male314 x Female334 | 5.97 | 3.08 |
| Male314 x Female338 | 4.98 | 1.72 |
| Male314 x Female347 | 4.83 | 1.52 |
| Male314 x Female349 | 6.26 | 2.12 |
| Male314 x Female351 | 1.44 | 2.68 |
| Male314 x Female355 | 4.14 | 2.17 |
| Male314 x Female357 | 4.84 | 1.69 |
| Male314 x Female358 | 4.84 | 1.93 |
| Male314 x Female360 | 5.78 | 3.08 |
| Male314 x Female367 | 2.26 | 2.59 |
| Male314 x Female383 | 2.58 | 2.65 |
| Male314 x Female386 | 1.45 | 2.40 |
| Male314 x Female388 | 1.35 | 3.18 |
| Male314 x Female392 | 5.89 | 3.16 |
| Male314 x Female396 | 4.08 | 1.65 |
| Male314 x Female402 | 6.12 | 2.50 |
| Male314 x Female407 | 1.60 | 3.14 |
| Male314 x Female415 | 2.69 | 3.37 |
| Male314 x Female419 | 2.43 | 1.43 |
| Male314 x Female424 | 3.00 | 3.30 |
| Male314 x Female429 | 4.46 | 2.11 |
| Male314 x Female432 | 5.31 | 2.00 |
| Male314 x Female436 | 4.38 | 3.30 |
| Male314 x Female446 | 5.77 | 1.69 |
| Male314 x Female448 | 4.07 | 1.79 |
| Male314 x Female451 | 5.37 | 2.53 |
| Male314 x Female456 | 5.57 | 1.43 |
| Male314 x Female459 | 1.75 | 1.63 |
| Male314 x Female461 | 4.57 | 1.61 |
| Male314 x Female463 | 4.30 | 2.23 |
| Male314 x Female467 | 6.12 | 2.70 |
| Male314 x Female470 | 2.34 | 2.07 |
| Male314 x Female473 | 2.12 | 2.72 |
| Male314 x Female480 | 1.40 | 1.53 |
| Male314 x Female482 | 1.92 | 2.56 |
| Male314 x Female489 | 3.70 | 2.18 |
| Male314 x Female494 | 2.66 | 2.86 |
| Male314 x Female500 | 4.01 | 2.09 |
| Male315 x Female302 | 4.56 | 2.91 |
| Male315 x Female306 | 5.47 | 2.28 |
| Male315 x Female315 | 3.42 | 2.44 |
| Male315 x Female317 | 4.71 | 1.83 |
| Male315 x Female322 | 5.31 | 2.81 |
| Male315 x Female324 | 2.95 | 2.11 |
| Male315 x Female330 | 3.39 | 2.26 |
| Male315 x Female333 | 4.81 | 2.80 |
| Male315 x Female335 | 3.39 | 1.93 |
| Male315 x Female337 | 5.65 | 1.79 |
| Male315 x Female344 | 4.20 | 1.48 |
| Male315 x Female348 | 4.57 | 2.00 |
| Male315 x Female363 | 2.69 | 2.01 |
| Male315 x Female370 | 2.69 | 1.85 |
| Male315 x Female377 | 4.92 | 2.77 |
| Male315 x Female384 | 3.51 | 1.53 |
| Male315 x Female387 | 2.46 | 1.61 |
| Male315 x Female389 | 1.35 | 4.05 |
| Male315 x Female395 | 6.40 | 1.42 |
| Male315 x Female397 | 5.44 | 1.86 |
| Male315 x Female401 | 5.80 | 1.79 |
| Male315 x Female404 | 5.45 | 2.03 |
| Male315 x Female414 | 5.47 | 1.38 |
| Male315 x Female418 | 5.27 | 2.54 |
| Male315 x Female420 | 2.89 | 1.88 |
| Male315 x Female422 | 2.52 | 3.00 |
| Male315 x Female425 | 3.28 | 1.52 |
| Male315 x Female431 | 3.46 | 2.76 |
| Male315 x Female434 | 4.53 | 2.02 |
| Male315 x Female445 | 5.05 | 2.62 |
| Male315 x Female450 | 5.32 | 2.00 |
| Male315 x Female460 | 3.33 | 1.93 |
| Male315 x Female462 | 5.65 | 1.88 |
| Male315 x Female466 | 2.29 | 1.99 |
| Male315 x Female472 | 3.33 | 2.26 |
| Male315 x Female477 | 2.08 | 2.75 |
| Male315 x Female481 | 6.55 | 2.71 |
| Male315 x Female483 | 3.24 | 1.38 |
| Male315 x Female493 | 4.51 | 3.25 |
| Male315 x Female499 | 3.58 | 1.76 |
| Male316 x Female302 | 4.86 | 3.45 |
| Male316 x Female306 | 3.47 | 3.32 |
| Male316 x Female315 | 3.94 | 3.14 |
| Male316 x Female317 | 5.99 | 2.81 |
| Male316 x Female322 | 5.45 | 3.95 |
| Male316 x Female324 | 2.47 | 3.23 |
| Male316 x Female330 | 5.01 | 2.88 |
| Male316 x Female335 | 2.96 | 1.64 |
| Male316 x Female337 | 5.82 | 2.47 |
| Male316 x Female344 | 4.77 | 2.67 |
| Male316 x Female348 | 4.95 | 1.83 |
| Male316 x Female363 | 3.49 | 1.82 |
| Male316 x Female370 | 4.81 | 1.64 |
| Male316 x Female377 | 5.23 | 3.76 |
| Male316 x Female384 | 2.83 | 1.35 |
| Male316 x Female387 | 2.09 | 1.31 |
| Male316 x Female389 | 1.84 | 3.82 |
| Male316 x Female395 | 5.34 | 2.12 |
| Male316 x Female397 | 3.72 | 2.49 |
| Male316 x Female401 | 5.27 | 1.69 |
| Male316 x Female404 | 5.35 | 1.74 |
| Male316 x Female418 | 5.64 | 3.13 |
| Male316 x Female420 | 1.36 | 2.19 |
| Male316 x Female422 | 2.74 | 2.89 |
| Male316 x Female425 | 3.19 | 2.79 |
| Male316 x Female431 | 4.52 | 2.99 |
| Male316 x Female434 | 5.72 | 1.81 |
| Male316 x Female445 | 5.76 | 2.52 |
| Male316 x Female450 | 4.66 | 1.62 |
| Male316 x Female454 | 2.47 | 3.71 |
| Male316 x Female457 | 2.54 | 4.57 |
| Male316 x Female460 | 2.85 | 3.19 |
| Male316 x Female462 | 3.15 | 5.33 |
| Male316 x Female466 | 3.93 | 4.02 |
| Male316 x Female468 | 1.62 | 1.78 |
| Male316 x Female472 | 2.74 | 3.90 |
| Male316 x Female477 | 2.04 | 1.56 |
| Male316 x Female481 | 5.89 | 4.40 |
| Male316 x Female483 | 2.63 | 2.23 |
| Male316 x Female499 | 4.60 | 1.76 |
| Male317 x Female305 | 6.03 | 2.31 |
| Male317 x Female316 | 4.51 | 1.87 |
| Male317 x Female323 | 1.45 | 1.53 |
| Male317 x Female327 | 2.53 | 2.02 |
| Male317 x Female332 | 3.52 | 1.51 |
| Male317 x Female334 | 4.84 | 2.28 |
| Male317 x Female338 | 4.18 | 1.67 |
| Male317 x Female347 | 4.36 | 2.59 |
| Male317 x Female349 | 4.35 | 1.30 |
| Male317 x Female351 | 1.32 | 2.55 |
| Male317 x Female355 | 3.57 | 1.78 |
| Male317 x Female357 | 4.26 | 2.10 |
| Male317 x Female358 | 4.31 | 1.47 |
| Male317 x Female360 | 6.31 | 3.66 |
| Male317 x Female367 | 1.45 | 1.93 |
| Male317 x Female386 | 1.88 | 4.12 |
| Male317 x Female388 | 0.92 | 3.47 |
| Male317 x Female396 | 4.43 | 1.34 |
| Male317 x Female402 | 3.22 | 2.18 |
| Male317 x Female407 | 2.26 | 2.38 |
| Male317 x Female415 | 2.45 | 4.00 |
| Male317 x Female424 | 1.31 | 1.36 |
| Male317 x Female432 | 4.51 | 1.69 |
| Male317 x Female436 | 3.88 | 2.00 |
| Male317 x Female448 | 2.43 | 1.46 |
| Male317 x Female451 | 4.49 | 2.18 |
| Male317 x Female456 | 4.85 | 1.90 |
| Male317 x Female459 | 1.78 | 1.39 |
| Male317 x Female461 | 4.02 | 2.00 |
| Male317 x Female463 | 3.94 | 2.53 |
| Male317 x Female470 | 2.09 | 2.12 |
| Male317 x Female473 | 0.85 | 1.35 |
| Male317 x Female480 | 1.50 | 2.04 |
| Male317 x Female482 | 1.83 | 1.93 |
| Male317 x Female494 | 3.16 | 1.96 |
| Male317 x Female500 | 3.85 | 1.94 |
| Male318 x Female302 | 5.36 | 3.03 |
| Male318 x Female306 | 3.74 | 2.36 |
| Male318 x Female315 | 3.82 | 1.64 |
| Male318 x Female317 | 5.01 | 2.59 |
| Male318 x Female322 | 4.20 | 2.73 |
| Male318 x Female324 | 1.63 | 2.63 |
| Male318 x Female330 | 4.99 | 1.99 |
| Male318 x Female335 | 2.40 | 1.44 |
| Male318 x Female337 | 5.11 | 2.37 |
| Male318 x Female344 | 3.55 | 2.28 |
| Male318 x Female348 | 5.30 | 2.22 |
| Male318 x Female363 | 1.88 | 1.76 |
| Male318 x Female370 | 2.06 | 1.40 |
| Male318 x Female377 | 5.49 | 2.12 |
| Male318 x Female384 | 2.35 | 1.50 |
| Male318 x Female387 | 2.92 | 1.76 |
| Male318 x Female389 | 1.26 | 3.08 |
| Male318 x Female395 | 5.17 | 1.93 |
| Male318 x Female397 | 4.89 | 2.13 |
| Male318 x Female401 | 3.25 | 2.33 |
| Male318 x Female404 | 4.45 | 1.29 |
| Male318 x Female414 | 4.65 | 2.29 |
| Male318 x Female418 | 5.18 | 2.51 |
| Male318 x Female420 | 1.49 | 1.76 |
| Male318 x Female422 | 2.46 | 2.08 |
| Male318 x Female425 | 3.41 | 1.96 |
| Male318 x Female431 | 3.94 | 2.09 |
| Male318 x Female434 | 4.51 | 2.24 |
| Male318 x Female445 | 3.99 | 2.26 |
| Male318 x Female447 | 3.56 | 3.03 |
| Male318 x Female450 | 4.12 | 2.04 |
| Male318 x Female454 | 2.33 | 3.03 |
| Male318 x Female457 | 1.92 | 3.49 |
| Male318 x Female460 | 2.15 | 2.76 |
| Male318 x Female462 | 2.94 | 2.23 |
| Male318 x Female466 | 3.56 | 1.82 |
| Male318 x Female468 | 1.47 | 1.85 |
| Male318 x Female472 | 1.77 | 2.80 |
| Male318 x Female477 | 1.98 | 1.72 |
| Male318 x Female481 | 5.52 | 3.25 |
| Male318 x Female483 | 2.13 | 1.88 |
| Male318 x Female493 | 4.84 | 2.43 |
| Male318 x Female499 | 3.66 | 1.62 |
| Male319 x Female305 | 4.70 | 4.02 |
| Male319 x Female314 | 1.87 | 1.38 |
| Male319 x Female316 | 5.45 | 2.20 |
| Male319 x Female320 | 5.11 | 2.63 |
| Male319 x Female323 | 1.75 | 1.77 |
| Male319 x Female325 | 3.02 | 1.86 |
| Male319 x Female327 | 3.08 | 2.63 |
| Male319 x Female332 | 4.97 | 3.12 |
| Male319 x Female334 | 4.16 | 2.28 |
| Male319 x Female338 | 5.18 | 1.73 |
| Male319 x Female347 | 5.82 | 2.43 |
| Male319 x Female349 | 4.66 | 2.22 |
| Male319 x Female351 | 1.17 | 3.71 |
| Male319 x Female355 | 2.81 | 1.51 |
| Male319 x Female357 | 3.61 | 2.16 |
| Male319 x Female358 | 4.82 | 2.20 |
| Male319 x Female360 | 6.44 | 3.42 |
| Male319 x Female367 | 1.36 | 2.59 |
| Male319 x Female383 | 2.95 | 4.21 |
| Male319 x Female386 | 1.24 | 4.52 |
| Male319 x Female388 | 1.30 | 3.75 |
| Male319 x Female392 | 5.15 | 1.44 |
| Male319 x Female396 | 5.58 | 1.91 |
| Male319 x Female402 | 2.88 | 2.97 |
| Male319 x Female407 | 2.31 | 2.90 |
| Male319 x Female415 | 3.34 | 4.51 |
| Male319 x Female417 | 4.79 | 4.24 |
| Male319 x Female419 | 2.21 | 1.79 |
| Male319 x Female424 | 3.47 | 2.17 |
| Male319 x Female429 | 4.64 | 2.68 |
| Male319 x Female432 | 5.30 | 1.64 |
| Male319 x Female436 | 4.07 | 2.22 |
| Male319 x Female446 | 4.49 | 3.46 |
| Male319 x Female448 | 3.23 | 1.51 |
| Male319 x Female451 | 4.20 | 2.52 |
| Male319 x Female456 | 6.18 | 1.47 |
| Male319 x Female459 | 1.18 | 1.95 |
| Male319 x Female461 | 3.86 | 2.86 |
| Male319 x Female463 | 3.44 | 2.29 |
| Male319 x Female467 | 5.55 | 1.93 |
| Male319 x Female470 | 1.32 | 1.90 |
| Male319 x Female473 | 1.26 | 2.29 |
| Male319 x Female480 | 1.02 | 1.52 |
| Male319 x Female482 | 2.06 | 3.29 |
| Male319 x Female489 | 5.10 | 1.78 |
| Male319 x Female494 | 1.94 | 3.10 |
| Male319 x Female498 | 2.30 | 2.42 |
| Male319 x Female500 | 3.04 | 2.06 |
| Male320 x Female302 | 6.66 | 1.98 |
| Male320 x Female306 | 5.88 | 2.45 |
| Male320 x Female315 | 2.71 | 1.50 |
| Male320 x Female317 | 4.79 | 1.76 |
| Male320 x Female322 | 6.08 | 2.91 |
| Male320 x Female324 | 3.08 | 1.16 |
| Male320 x Female326 | 6.80 | 1.06 |
| Male320 x Female330 | 5.19 | 2.20 |
| Male320 x Female333 | 5.66 | 1.89 |
| Male320 x Female335 | 3.15 | 1.65 |
| Male320 x Female337 | 5.95 | 1.55 |
| Male320 x Female344 | 5.04 | 2.00 |
| Male320 x Female348 | 5.28 | 2.72 |
| Male320 x Female353 | 1.80 | 2.76 |
| Male320 x Female363 | 4.20 | 1.95 |
| Male320 x Female370 | 3.45 | 1.22 |
| Male320 x Female377 | 6.14 | 1.39 |
| Male320 x Female384 | 4.13 | 1.67 |
| Male320 x Female387 | 3.94 | 1.49 |
| Male320 x Female389 | 1.37 | 1.58 |
| Male320 x Female395 | 6.50 | 2.33 |
| Male320 x Female397 | 4.69 | 1.39 |
| Male320 x Female401 | 6.47 | 2.45 |
| Male320 x Female404 | 6.41 | 3.01 |
| Male320 x Female414 | 5.37 | 1.69 |
| Male320 x Female416 | 2.41 | 1.36 |
| Male320 x Female418 | 5.80 | 1.91 |
| Male320 x Female420 | 1.68 | 1.34 |
| Male320 x Female422 | 3.26 | 1.84 |
| Male320 x Female425 | 4.00 | 1.90 |
| Male320 x Female431 | 4.12 | 1.60 |
| Male320 x Female434 | 5.51 | 1.69 |
| Male320 x Female445 | 6.19 | 2.64 |
| Male320 x Female447 | 4.02 | 2.46 |
| Male320 x Female450 | 5.73 | 2.05 |
| Male320 x Female454 | 2.63 | 1.73 |
| Male320 x Female457 | 3.33 | 1.76 |
| Male320 x Female460 | 3.78 | 1.98 |
| Male320 x Female462 | 4.98 | 1.94 |
| Male320 x Female466 | 3.22 | 1.33 |
| Male320 x Female468 | 1.94 | 1.32 |
| Male320 x Female472 | 2.25 | 2.33 |
| Male320 x Female477 | 2.41 | 1.39 |
| Male320 x Female481 | 6.36 | 2.26 |
| Male320 x Female483 | 3.09 | 1.30 |
| Male320 x Female493 | 5.75 | 1.58 |
| Male320 x Female499 | 3.85 | 1.55 |
| Male321 x Female302 | 5.46 | 3.41 |
| Male321 x Female306 | 3.75 | 2.26 |
| Male321 x Female315 | 4.01 | 2.88 |
| Male321 x Female317 | 5.26 | 2.22 |
| Male321 x Female322 | 5.47 | 3.12 |
| Male321 x Female324 | 3.47 | 1.60 |
| Male321 x Female326 | 5.58 | 2.44 |
| Male321 x Female330 | 5.10 | 2.42 |
| Male321 x Female333 | 5.20 | 2.26 |
| Male321 x Female335 | 2.32 | 1.53 |
| Male321 x Female337 | 5.59 | 1.98 |
| Male321 x Female344 | 4.73 | 2.66 |
| Male321 x Female348 | 4.77 | 2.63 |
| Male321 x Female350 | 1.42 | 1.39 |
| Male321 x Female353 | 2.06 | 1.96 |
| Male321 x Female363 | 2.12 | 1.52 |
| Male321 x Female370 | 2.50 | 1.53 |
| Male321 x Female377 | 5.27 | 1.60 |
| Male321 x Female384 | 2.53 | 1.35 |
| Male321 x Female387 | 3.18 | 1.66 |
| Male321 x Female389 | 1.67 | 2.11 |
| Male321 x Female395 | 5.97 | 1.60 |
| Male321 x Female397 | 4.19 | 2.15 |
| Male321 x Female401 | 3.93 | 1.99 |
| Male321 x Female404 | 5.79 | 2.26 |
| Male321 x Female414 | 4.55 | 2.30 |
| Male321 x Female416 | 2.22 | 1.76 |
| Male321 x Female418 | 5.21 | 2.51 |
| Male321 x Female420 | 1.56 | 1.51 |
| Male321 x Female422 | 3.02 | 1.61 |
| Male321 x Female425 | 3.87 | 1.89 |
| Male321 x Female431 | 4.52 | 2.04 |
| Male321 x Female434 | 5.12 | 1.89 |
| Male321 x Female445 | 5.37 | 2.09 |
| Male321 x Female447 | 5.23 | 3.38 |
| Male321 x Female450 | 3.68 | 2.28 |
| Male321 x Female454 | 2.59 | 1.79 |
| Male321 x Female457 | 3.33 | 2.20 |
| Male321 x Female460 | 3.09 | 2.02 |
| Male321 x Female462 | 6.62 | 2.28 |
| Male321 x Female466 | 3.34 | 1.97 |
| Male321 x Female468 | 1.97 | 1.46 |
| Male321 x Female472 | 4.44 | 1.75 |
| Male321 x Female477 | 1.80 | 1.48 |
| Male321 x Female479 | 2.73 | 1.78 |
| Male321 x Female481 | 5.63 | 2.32 |
| Male321 x Female483 | 2.89 | 1.23 |
| Male321 x Female493 | 5.28 | 2.86 |
| Male321 x Female499 | 4.00 | 1.78 |
| Male322 x Female305 | 6.09 | 3.48 |
| Male322 x Female314 | 1.11 | 1.23 |
| Male322 x Female316 | 3.16 | 2.57 |
| Male322 x Female320 | 5.85 | 2.01 |
| Male322 x Female323 | 2.14 | 1.67 |
| Male322 x Female325 | 2.71 | 1.28 |
| Male322 x Female327 | 3.07 | 2.40 |
| Male322 x Female332 | 4.96 | 1.62 |
| Male322 x Female334 | 4.92 | 2.17 |
| Male322 x Female338 | 5.07 | 2.10 |
| Male322 x Female347 | 5.64 | 3.14 |
| Male322 x Female349 | 4.34 | 1.80 |
| Male322 x Female351 | 1.65 | 3.15 |
| Male322 x Female355 | 3.38 | 1.65 |
| Male322 x Female357 | 4.11 | 3.18 |
| Male322 x Female358 | 5.01 | 1.64 |
| Male322 x Female360 | 6.20 | 2.22 |
| Male322 x Female367 | 1.33 | 2.31 |
| Male322 x Female383 | 3.38 | 1.39 |
| Male322 x Female386 | 1.54 | 2.36 |
| Male322 x Female388 | 0.90 | 1.98 |
| Male322 x Female392 | 5.16 | 1.73 |
| Male322 x Female396 | 4.58 | 2.34 |
| Male322 x Female402 | 3.36 | 2.48 |
| Male322 x Female407 | 2.35 | 2.06 |
| Male322 x Female415 | 2.05 | 2.92 |
| Male322 x Female419 | 2.79 | 1.60 |
| Male322 x Female424 | 2.39 | 1.21 |
| Male322 x Female429 | 4.38 | 1.99 |
| Male322 x Female432 | 5.30 | 1.27 |
| Male322 x Female436 | 3.45 | 2.13 |
| Male322 x Female446 | 5.15 | 2.37 |
| Male322 x Female448 | 3.05 | 1.57 |
| Male322 x Female451 | 4.45 | 2.46 |
| Male322 x Female456 | 6.13 | 2.13 |
| Male322 x Female459 | 2.79 | 1.78 |
| Male322 x Female461 | 3.84 | 1.86 |
| Male322 x Female463 | 5.44 | 2.39 |
| Male322 x Female467 | 5.18 | 1.64 |
| Male322 x Female470 | 2.24 | 2.14 |
| Male322 x Female473 | 1.62 | 2.22 |
| Male322 x Female480 | 1.42 | 1.55 |
| Male322 x Female482 | 2.54 | 2.52 |
| Male322 x Female489 | 4.95 | 2.94 |
| Male322 x Female494 | 3.30 | 1.81 |
| Male322 x Female498 | 2.11 | 1.75 |
| Male322 x Female500 | 4.30 | 1.44 |
| Male323 x Female302 | 3.64 | 1.99 |
| Male323 x Female306 | 2.51 | 1.49 |
| Male323 x Female315 | 1.85 | 1.86 |
| Male323 x Female317 | 1.63 | 1.44 |
| Male323 x Female322 | 2.72 | 1.75 |
| Male323 x Female324 | 3.01 | 1.84 |
| Male323 x Female330 | 3.39 | 1.32 |
| Male323 x Female335 | 2.70 | 1.44 |
| Male323 x Female337 | 3.16 | 1.55 |
| Male323 x Female344 | 2.07 | 1.45 |
| Male323 x Female348 | 2.97 | 1.85 |
| Male323 x Female363 | 2.48 | 1.48 |
| Male323 x Female370 | 2.25 | 1.69 |
| Male323 x Female384 | 2.45 | 1.22 |
| Male323 x Female387 | 5.82 | 2.54 |
| Male323 x Female389 | 0.94 | 1.55 |
| Male323 x Female395 | 3.42 | 1.50 |
| Male323 x Female401 | 1.66 | 1.90 |
| Male323 x Female404 | 4.00 | 1.36 |
| Male323 x Female420 | 1.62 | 1.85 |
| Male323 x Female422 | 2.69 | 1.48 |
| Male323 x Female425 | 2.69 | 2.00 |
| Male323 x Female431 | 3.21 | 1.30 |
| Male323 x Female434 | 3.48 | 1.91 |
| Male323 x Female445 | 2.37 | 2.01 |
| Male323 x Female450 | 1.96 | 1.44 |
| Male323 x Female457 | 1.53 | 1.37 |
| Male323 x Female460 | 2.38 | 1.83 |
| Male323 x Female462 | 2.29 | 1.48 |
| Male323 x Female466 | 2.62 | 1.85 |
| Male323 x Female472 | 2.32 | 1.68 |
| Male323 x Female477 | 2.43 | 1.16 |
| Male323 x Female481 | 2.48 | 1.67 |
| Male323 x Female483 | 2.67 | 1.85 |
| Male323 x Female493 | 2.39 | 2.03 |
| Male323 x Female499 | 3.00 | 1.40 |
| Male324 x Female305 | 5.80 | 3.75 |
| Male324 x Female316 | 4.36 | 2.00 |
| Male324 x Female323 | 1.96 | 1.42 |
| Male324 x Female327 | 2.24 | 2.53 |
| Male324 x Female332 | 5.38 | 2.08 |
| Male324 x Female334 | 5.18 | 1.64 |
| Male324 x Female338 | 5.78 | 1.92 |
| Male324 x Female347 | 4.70 | 2.83 |
| Male324 x Female349 | 5.28 | 2.57 |
| Male324 x Female351 | 0.90 | 2.27 |
| Male324 x Female355 | 4.36 | 1.80 |
| Male324 x Female357 | 4.26 | 2.08 |
| Male324 x Female358 | 5.35 | 2.00 |
| Male324 x Female360 | 5.48 | 2.47 |
| Male324 x Female367 | 1.85 | 2.07 |
| Male324 x Female383 | 4.13 | 2.74 |
| Male324 x Female388 | 1.38 | 3.47 |
| Male324 x Female392 | 6.24 | 1.94 |
| Male324 x Female396 | 4.50 | 2.11 |
| Male324 x Female402 | 4.70 | 3.30 |
| Male324 x Female407 | 2.00 | 2.31 |
| Male324 x Female415 | 3.23 | 3.82 |
| Male324 x Female419 | 2.70 | 1.45 |
| Male324 x Female424 | 3.58 | 2.14 |
| Male324 x Female429 | 4.92 | 1.61 |
| Male324 x Female432 | 6.43 | 1.72 |
| Male324 x Female436 | 4.92 | 2.81 |
| Male324 x Female446 | 5.92 | 2.69 |
| Male324 x Female448 | 3.83 | 1.46 |
| Male324 x Female451 | 4.88 | 2.38 |
| Male324 x Female456 | 5.73 | 1.54 |
| Male324 x Female459 | 2.93 | 1.53 |
| Male324 x Female461 | 4.40 | 2.18 |
| Male324 x Female463 | 5.61 | 2.28 |
| Male324 x Female467 | 5.38 | 1.98 |
| Male324 x Female470 | 2.14 | 1.62 |
| Male324 x Female473 | 2.32 | 2.64 |
| Male324 x Female480 | 1.30 | 1.38 |
| Male324 x Female494 | 3.18 | 2.10 |
| Male324 x Female498 | 2.13 | 2.50 |
| Male324 x Female500 | 5.47 | 1.80 |
| Male325 x Female302 | 3.47 | 3.66 |
| Male325 x Female306 | 1.93 | 2.28 |
| Male325 x Female315 | 2.18 | 2.56 |
| Male325 x Female317 | 2.32 | 2.51 |
| Male325 x Female322 | 3.13 | 3.00 |
| Male325 x Female324 | 2.30 | 2.10 |
| Male325 x Female330 | 1.83 | 2.52 |
| Male325 x Female333 | 2.63 | 4.91 |
| Male325 x Female335 | 1.95 | 1.53 |
| Male325 x Female337 | 2.91 | 3.04 |
| Male325 x Female344 | 1.66 | 2.06 |
| Male325 x Female348 | 1.84 | 1.66 |
| Male325 x Female363 | 1.72 | 1.49 |
| Male325 x Female370 | 2.09 | 2.05 |
| Male325 x Female377 | 2.69 | 2.35 |
| Male325 x Female384 | 1.87 | 2.30 |
| Male325 x Female387 | 1.82 | 1.45 |
| Male325 x Female389 | 2.14 | 3.56 |
| Male325 x Female395 | 2.53 | 1.37 |
| Male325 x Female397 | 2.97 | 2.83 |
| Male325 x Female401 | 1.90 | 2.14 |
| Male325 x Female404 | 3.41 | 1.91 |
| Male325 x Female414 | 1.24 | 2.40 |
| Male325 x Female416 | 1.40 | 2.82 |
| Male325 x Female418 | 1.73 | 2.09 |
| Male325 x Female420 | 1.27 | 1.81 |
| Male325 x Female422 | 2.03 | 3.19 |
| Male325 x Female425 | 2.92 | 3.93 |
| Male325 x Female431 | 2.57 | 2.09 |
| Male325 x Female434 | 3.47 | 1.81 |
| Male325 x Female445 | 3.65 | 2.21 |
| Male325 x Female447 | 2.26 | 4.84 |
| Male325 x Female450 | 2.65 | 1.88 |
| Male325 x Female454 | 1.72 | 3.50 |
| Male325 x Female457 | 2.20 | 4.54 |
| Male325 x Female460 | 2.72 | 3.17 |
| Male325 x Female462 | 2.54 | 4.90 |
| Male325 x Female466 | 1.50 | 2.22 |
| Male325 x Female468 | 2.18 | 1.37 |
| Male325 x Female472 | 2.20 | 2.09 |
| Male325 x Female477 | 1.35 | 1.60 |
| Male325 x Female481 | 3.78 | 2.23 |
| Male325 x Female483 | 2.34 | 1.85 |
| Male325 x Female493 | 2.24 | 3.37 |
| Male325 x Female499 | 3.92 | 1.60 |
| Male326 x Female305 | 5.50 | 2.61 |
| Male326 x Female316 | 4.62 | 1.93 |
| Male326 x Female320 | 4.42 | 1.91 |
| Male326 x Female323 | 1.87 | 2.26 |
| Male326 x Female325 | 2.54 | 2.35 |
| Male326 x Female327 | 2.98 | 2.59 |
| Male326 x Female332 | 5.37 | 3.74 |
| Male326 x Female334 | 4.82 | 2.78 |
| Male326 x Female336 | 3.87 | 2.68 |
| Male326 x Female338 | 5.57 | 1.92 |
| Male326 x Female347 | 4.56 | 2.96 |
| Male326 x Female349 | 4.95 | 2.72 |
| Male326 x Female351 | 1.62 | 2.74 |
| Male326 x Female355 | 3.69 | 2.11 |
| Male326 x Female357 | 5.19 | 1.99 |
| Male326 x Female358 | 4.75 | 1.49 |
| Male326 x Female360 | 5.62 | 2.15 |
| Male326 x Female367 | 2.28 | 2.36 |
| Male326 x Female383 | 3.30 | 3.29 |
| Male326 x Female386 | 1.41 | 3.94 |
| Male326 x Female388 | 2.56 | 4.05 |
| Male326 x Female392 | 5.64 | 1.45 |
| Male326 x Female396 | 5.21 | 1.44 |
| Male326 x Female402 | 2.71 | 2.31 |
| Male326 x Female407 | 2.49 | 2.54 |
| Male326 x Female415 | 2.25 | 4.78 |
| Male326 x Female419 | 2.42 | 1.67 |
| Male326 x Female424 | 2.31 | 2.34 |
| Male326 x Female429 | 3.95 | 2.84 |
| Male326 x Female432 | 4.82 | 1.94 |
| Male326 x Female436 | 4.54 | 1.58 |
| Male326 x Female446 | 5.24 | 2.51 |
| Male326 x Female448 | 2.83 | 1.72 |
| Male326 x Female451 | 5.92 | 2.25 |
| Male326 x Female456 | 5.59 | 1.81 |
| Male326 x Female459 | 1.28 | 1.48 |
| Male326 x Female461 | 4.30 | 1.89 |
| Male326 x Female463 | 3.11 | 2.03 |
| Male326 x Female467 | 4.94 | 1.61 |
| Male326 x Female470 | 2.06 | 2.85 |
| Male326 x Female473 | 2.23 | 3.44 |
| Male326 x Female480 | 1.37 | 1.22 |
| Male326 x Female482 | 1.97 | 2.70 |
| Male326 x Female494 | 3.54 | 2.35 |
| Male326 x Female498 | 2.42 | 2.60 |
| Male326 x Female500 | 4.35 | 2.40 |
| Male327 x Female302 | 6.13 | 2.70 |
| Male327 x Female306 | 3.15 | 2.38 |
| Male327 x Female315 | 3.07 | 3.13 |
| Male327 x Female317 | 4.04 | 2.65 |
| Male327 x Female322 | 4.75 | 2.48 |
| Male327 x Female324 | 3.20 | 1.93 |
| Male327 x Female326 | 2.37 | 3.36 |
| Male327 x Female330 | 3.41 | 2.73 |
| Male327 x Female335 | 2.62 | 2.01 |
| Male327 x Female337 | 3.28 | 2.24 |
| Male327 x Female344 | 2.65 | 1.71 |
| Male327 x Female348 | 2.80 | 1.73 |
| Male327 x Female363 | 2.53 | 3.51 |
| Male327 x Female370 | 3.18 | 1.48 |
| Male327 x Female384 | 3.22 | 1.81 |
| Male327 x Female387 | 2.98 | 1.32 |
| Male327 x Female389 | 2.51 | 3.36 |
| Male327 x Female395 | 3.34 | 1.90 |
| Male327 x Female397 | 3.24 | 1.97 |
| Male327 x Female401 | 3.15 | 1.68 |
| Male327 x Female404 | 4.83 | 1.78 |
| Male327 x Female414 | 2.58 | 1.57 |
| Male327 x Female418 | 2.84 | 2.30 |
| Male327 x Female420 | 1.88 | 1.41 |
| Male327 x Female422 | 2.32 | 2.56 |
| Male327 x Female425 | 2.16 | 1.82 |
| Male327 x Female431 | 2.85 | 2.04 |
| Male327 x Female434 | 3.10 | 1.75 |
| Male327 x Female445 | 3.33 | 2.02 |
| Male327 x Female450 | 2.65 | 1.57 |
| Male327 x Female460 | 2.79 | 1.97 |
| Male327 x Female462 | 3.00 | 3.77 |
| Male327 x Female466 | 3.44 | 4.07 |
| Male327 x Female472 | 3.59 | 4.47 |
| Male327 x Female477 | 2.83 | 1.63 |
| Male327 x Female481 | 4.59 | 2.43 |
| Male327 x Female483 | 2.40 | 1.75 |
| Male327 x Female493 | 2.97 | 2.95 |
| Male327 x Female499 | 4.21 | 1.29 |
| Male328 x Female302 | 5.98 | 3.32 |
| Male328 x Female306 | 3.46 | 3.03 |
| Male328 x Female315 | 3.19 | 2.40 |
| Male328 x Female317 | 5.32 | 2.01 |
| Male328 x Female322 | 5.37 | 3.15 |
| Male328 x Female324 | 2.28 | 3.53 |
| Male328 x Female330 | 4.53 | 2.87 |
| Male328 x Female335 | 2.95 | 1.22 |
| Male328 x Female337 | 5.10 | 2.33 |
| Male328 x Female344 | 4.30 | 1.40 |
| Male328 x Female348 | 4.41 | 1.88 |
| Male328 x Female363 | 3.10 | 1.78 |
| Male328 x Female370 | 2.66 | 1.52 |
| Male328 x Female384 | 2.44 | 1.55 |
| Male328 x Female387 | 4.27 | 2.05 |
| Male328 x Female389 | 1.44 | 2.90 |
| Male328 x Female395 | 5.60 | 1.53 |
| Male328 x Female397 | 4.64 | 2.38 |
| Male328 x Female401 | 4.33 | 2.25 |
| Male328 x Female404 | 4.23 | 1.97 |
| Male328 x Female414 | 4.24 | 2.80 |
| Male328 x Female418 | 4.56 | 2.45 |
| Male328 x Female420 | 1.60 | 1.44 |
| Male328 x Female422 | 2.64 | 2.03 |
| Male328 x Female425 | 3.20 | 2.25 |
| Male328 x Female431 | 4.39 | 3.17 |
| Male328 x Female434 | 5.11 | 1.56 |
| Male328 x Female445 | 5.32 | 3.16 |
| Male328 x Female450 | 4.11 | 1.43 |
| Male328 x Female454 | 1.61 | 2.19 |
| Male328 x Female457 | 1.97 | 2.93 |
| Male328 x Female460 | 2.10 | 1.92 |
| Male328 x Female462 | 3.14 | 2.26 |
| Male328 x Female466 | 3.08 | 2.71 |
| Male328 x Female468 | 2.38 | 1.64 |
| Male328 x Female472 | 3.25 | 2.87 |
| Male328 x Female477 | 1.86 | 2.16 |
| Male328 x Female481 | 5.75 | 3.30 |
| Male328 x Female483 | 2.48 | 2.25 |
| Male328 x Female493 | 4.95 | 2.94 |
| Male328 x Female499 | 3.59 | 1.64 |
| Male329 x Female305 | 6.00 | 2.39 |
| Male329 x Female314 | 1.36 | 1.45 |
| Male329 x Female316 | 4.27 | 1.96 |
| Male329 x Female320 | 6.56 | 2.42 |
| Male329 x Female323 | 2.20 | 2.09 |
| Male329 x Female325 | 3.06 | 2.36 |
| Male329 x Female327 | 2.90 | 2.43 |
| Male329 x Female332 | 5.07 | 2.63 |
| Male329 x Female334 | 4.25 | 2.45 |
| Male329 x Female336 | 3.44 | 2.68 |
| Male329 x Female338 | 5.34 | 2.16 |
| Male329 x Female347 | 6.15 | 2.41 |
| Male329 x Female349 | 5.91 | 1.53 |
| Male329 x Female351 | 0.91 | 2.57 |
| Male329 x Female355 | 3.91 | 1.65 |
| Male329 x Female357 | 4.87 | 1.88 |
| Male329 x Female358 | 4.11 | 1.97 |
| Male329 x Female360 | 6.60 | 1.88 |
| Male329 x Female367 | 1.46 | 2.41 |
| Male329 x Female383 | 2.80 | 3.70 |
| Male329 x Female386 | 1.08 | 3.74 |
| Male329 x Female388 | 2.65 | 2.28 |
| Male329 x Female392 | 6.27 | 1.48 |
| Male329 x Female396 | 4.81 | 1.77 |
| Male329 x Female402 | 3.08 | 2.19 |
| Male329 x Female407 | 2.38 | 1.92 |
| Male329 x Female415 | 3.23 | 3.69 |
| Male329 x Female417 | 4.35 | 3.64 |
| Male329 x Female419 | 2.01 | 1.44 |
| Male329 x Female424 | 2.63 | 2.22 |
| Male329 x Female429 | 4.12 | 1.57 |
| Male329 x Female432 | 5.94 | 1.58 |
| Male329 x Female436 | 4.96 | 1.94 |
| Male329 x Female446 | 5.13 | 2.36 |
| Male329 x Female448 | 3.45 | 1.22 |
| Male329 x Female451 | 5.03 | 1.83 |
| Male329 x Female456 | 6.05 | 1.31 |
| Male329 x Female459 | 2.08 | 1.58 |
| Male329 x Female461 | 3.62 | 2.22 |
| Male329 x Female463 | 5.06 | 2.26 |
| Male329 x Female467 | 4.42 | 2.52 |
| Male329 x Female470 | 2.39 | 1.61 |
| Male329 x Female473 | 1.51 | 2.84 |
| Male329 x Female480 | 1.91 | 1.55 |
| Male329 x Female482 | 3.34 | 2.89 |
| Male329 x Female489 | 4.59 | 1.61 |
| Male329 x Female494 | 2.76 | 1.70 |
| Male329 x Female498 | 2.73 | 2.24 |
| Male329 x Female500 | 4.45 | 1.66 |
| Male330 x Female302 | 6.56 | 2.44 |
| Male330 x Female306 | 3.31 | 1.98 |
| Male330 x Female315 | 3.79 | 2.02 |
| Male330 x Female317 | 4.78 | 2.26 |
| Male330 x Female322 | 5.55 | 3.31 |
| Male330 x Female324 | 3.34 | 2.42 |
| Male330 x Female330 | 4.71 | 2.16 |
| Male330 x Female333 | 5.05 | 3.01 |
| Male330 x Female335 | 2.75 | 2.02 |
| Male330 x Female337 | 6.68 | 2.40 |
| Male330 x Female344 | 2.41 | 1.86 |
| Male330 x Female348 | 5.24 | 2.13 |
| Male330 x Female353 | 2.40 | 2.76 |
| Male330 x Female363 | 2.56 | 1.81 |
| Male330 x Female370 | 2.79 | 1.96 |
| Male330 x Female377 | 5.64 | 2.11 |
| Male330 x Female384 | 3.18 | 1.54 |
| Male330 x Female389 | 1.59 | 2.66 |
| Male330 x Female395 | 5.83 | 1.58 |
| Male330 x Female397 | 4.26 | 1.69 |
| Male330 x Female401 | 2.96 | 1.32 |
| Male330 x Female404 | 5.61 | 3.02 |
| Male330 x Female414 | 5.72 | 2.71 |
| Male330 x Female416 | 2.83 | 1.23 |
| Male330 x Female418 | 4.99 | 2.27 |
| Male330 x Female420 | 1.20 | 1.61 |
| Male330 x Female422 | 3.76 | 2.64 |
| Male330 x Female425 | 4.19 | 1.44 |
| Male330 x Female431 | 2.78 | 1.47 |
| Male330 x Female434 | 3.95 | 1.48 |
| Male330 x Female445 | 5.91 | 2.64 |
| Male330 x Female450 | 5.44 | 1.65 |
| Male330 x Female460 | 2.74 | 1.99 |
| Male330 x Female462 | 5.40 | 1.92 |
| Male330 x Female466 | 2.82 | 2.19 |
| Male330 x Female472 | 3.47 | 2.77 |
| Male330 x Female477 | 1.10 | 1.61 |
| Male330 x Female481 | 5.75 | 2.16 |
| Male330 x Female483 | 2.62 | 1.31 |
| Male330 x Female493 | 5.72 | 2.90 |
| Male330 x Female499 | 4.20 | 1.38 |
| Male331 x Female305 | 6.33 | 1.97 |
| Male331 x Female316 | 4.65 | 2.03 |
| Male331 x Female320 | 4.91 | 2.85 |
| Male331 x Female323 | 1.06 | 1.71 |
| Male331 x Female327 | 2.35 | 1.85 |
| Male331 x Female332 | 5.00 | 1.45 |
| Male331 x Female334 | 4.30 | 2.41 |
| Male331 x Female338 | 4.26 | 1.49 |
| Male331 x Female347 | 5.05 | 2.34 |
| Male331 x Female351 | 1.24 | 2.77 |
| Male331 x Female355 | 3.22 | 1.80 |
| Male331 x Female357 | 3.64 | 2.41 |
| Male331 x Female358 | 4.41 | 1.72 |
| Male331 x Female360 | 5.25 | 2.55 |
| Male331 x Female367 | 1.44 | 1.85 |
| Male331 x Female383 | 3.07 | 1.75 |
| Male331 x Female386 | 1.66 | 3.62 |
| Male331 x Female388 | 1.80 | 2.22 |
| Male331 x Female392 | 4.79 | 1.49 |
| Male331 x Female396 | 4.27 | 2.23 |
| Male331 x Female402 | 3.96 | 1.71 |
| Male331 x Female415 | 2.63 | 2.57 |
| Male331 x Female419 | 2.35 | 1.37 |
| Male331 x Female424 | 3.43 | 1.94 |
| Male331 x Female432 | 5.85 | 2.00 |
| Male331 x Female436 | 4.27 | 1.88 |
| Male331 x Female446 | 5.38 | 2.16 |
| Male331 x Female448 | 2.98 | 2.02 |
| Male331 x Female451 | 5.18 | 2.16 |
| Male331 x Female456 | 5.10 | 1.88 |
| Male331 x Female459 | 1.08 | 1.68 |
| Male331 x Female461 | 3.51 | 1.91 |
| Male331 x Female463 | 3.35 | 2.88 |
| Male331 x Female467 | 3.29 | 2.14 |
| Male331 x Female470 | 1.87 | 1.66 |
| Male331 x Female473 | 1.16 | 2.28 |
| Male331 x Female480 | 1.21 | 1.55 |
| Male331 x Female482 | 2.63 | 2.03 |
| Male331 x Female489 | 4.66 | 1.65 |
| Male331 x Female494 | 3.21 | 1.40 |
| Male331 x Female498 | 2.27 | 2.24 |
| Male331 x Female500 | 4.27 | 1.69 |
| Male332 x Female305 | 5.95 | 2.58 |
| Male332 x Female316 | 6.02 | 1.68 |
| Male332 x Female320 | 5.60 | 1.59 |
| Male332 x Female323 | 2.27 | 1.51 |
| Male332 x Female327 | 1.42 | 1.33 |
| Male332 x Female332 | 5.73 | 2.22 |
| Male332 x Female334 | 6.73 | 2.26 |
| Male332 x Female336 | 5.83 | 2.60 |
| Male332 x Female338 | 5.25 | 1.49 |
| Male332 x Female347 | 5.59 | 1.94 |
| Male332 x Female349 | 5.40 | 2.46 |
| Male332 x Female351 | 1.54 | 1.86 |
| Male332 x Female355 | 4.24 | 2.50 |
| Male332 x Female357 | 4.47 | 2.84 |
| Male332 x Female358 | 6.40 | 1.64 |
| Male332 x Female360 | 6.63 | 2.68 |
| Male332 x Female367 | 1.41 | 1.34 |
| Male332 x Female383 | 4.37 | 1.76 |
| Male332 x Female386 | 0.88 | 1.77 |
| Male332 x Female388 | 1.18 | 1.98 |
| Male332 x Female392 | 5.51 | 1.89 |
| Male332 x Female396 | 5.09 | 1.62 |
| Male332 x Female402 | 6.72 | 1.77 |
| Male332 x Female407 | 3.35 | 2.07 |
| Male332 x Female415 | 2.83 | 2.26 |
| Male332 x Female417 | 5.36 | 1.82 |
| Male332 x Female419 | 2.96 | 1.57 |
| Male332 x Female424 | 2.88 | 2.05 |
| Male332 x Female429 | 5.17 | 1.65 |
| Male332 x Female432 | 6.27 | 1.44 |
| Male332 x Female436 | 5.12 | 1.96 |
| Male332 x Female446 | 5.74 | 2.49 |
| Male332 x Female448 | 3.60 | 1.34 |
| Male332 x Female451 | 5.35 | 1.97 |
| Male332 x Female456 | 6.36 | 2.24 |
| Male332 x Female459 | 1.83 | 1.40 |
| Male332 x Female461 | 5.36 | 2.22 |
| Male332 x Female463 | 5.03 | 2.11 |
| Male332 x Female467 | 5.16 | 1.78 |
| Male332 x Female470 | 2.78 | 1.28 |
| Male332 x Female473 | 1.89 | 1.91 |
| Male332 x Female480 | 1.74 | 1.97 |
| Male332 x Female482 | 3.47 | 1.37 |
| Male332 x Female489 | 4.52 | 2.29 |
| Male332 x Female494 | 4.14 | 1.81 |
| Male332 x Female498 | 2.39 | 1.88 |
| Male332 x Female500 | 4.08 | 1.61 |
| Male333 x Female302 | 5.90 | 2.13 |
| Male333 x Female306 | 4.14 | 1.73 |
| Male333 x Female315 | 2.72 | 2.05 |
| Male333 x Female317 | 5.10 | 1.86 |
| Male333 x Female322 | 6.54 | 2.16 |
| Male333 x Female324 | 2.99 | 1.54 |
| Male333 x Female330 | 4.88 | 1.96 |
| Male333 x Female335 | 2.49 | 1.11 |
| Male333 x Female337 | 6.06 | 2.13 |
| Male333 x Female344 | 5.65 | 2.48 |
| Male333 x Female348 | 5.13 | 1.91 |
| Male333 x Female363 | 2.25 | 1.39 |
| Male333 x Female370 | 2.64 | 1.67 |
| Male333 x Female384 | 3.46 | 1.64 |
| Male333 x Female389 | 2.47 | 2.75 |
| Male333 x Female395 | 5.92 | 1.36 |
| Male333 x Female397 | 4.52 | 1.92 |
| Male333 x Female401 | 3.81 | 2.26 |
| Male333 x Female404 | 5.84 | 1.74 |
| Male333 x Female414 | 4.18 | 1.67 |
| Male333 x Female418 | 5.82 | 1.55 |
| Male333 x Female420 | 1.53 | 1.83 |
| Male333 x Female422 | 2.71 | 2.56 |
| Male333 x Female425 | 3.67 | 1.38 |
| Male333 x Female431 | 5.51 | 2.48 |
| Male333 x Female434 | 4.89 | 1.32 |
| Male333 x Female445 | 7.03 | 2.74 |
| Male333 x Female450 | 5.97 | 1.61 |
| Male333 x Female454 | 2.34 | 2.40 |
| Male333 x Female457 | 1.63 | 3.01 |
| Male333 x Female460 | 2.03 | 2.06 |
| Male333 x Female462 | 3.32 | 1.66 |
| Male333 x Female466 | 1.17 | 2.50 |
| Male333 x Female468 | 1.70 | 1.30 |
| Male333 x Female472 | 2.78 | 3.15 |
| Male333 x Female477 | 1.74 | 1.36 |
| Male333 x Female481 | 6.40 | 2.47 |
| Male333 x Female483 | 2.36 | 1.59 |
| Male333 x Female493 | 5.44 | 2.63 |
| Male333 x Female499 | 2.90 | 1.42 |
| Male334 x Female305 | 6.64 | 3.27 |
| Male334 x Female316 | 6.39 | 2.29 |
| Male334 x Female320 | 5.77 | 2.38 |
| Male334 x Female323 | 3.77 | 2.48 |
| Male334 x Female327 | 3.69 | 2.58 |
| Male334 x Female332 | 7.04 | 2.93 |
| Male334 x Female334 | 6.93 | 2.40 |
| Male334 x Female338 | 6.71 | 2.31 |
| Male334 x Female347 | 5.89 | 3.73 |
| Male334 x Female349 | 4.92 | 1.91 |
| Male334 x Female351 | 1.78 | 3.74 |
| Male334 x Female355 | 5.18 | 1.59 |
| Male334 x Female357 | 5.94 | 2.50 |
| Male334 x Female358 | 6.27 | 2.14 |
| Male334 x Female360 | 6.70 | 2.28 |
| Male334 x Female367 | 2.02 | 2.05 |
| Male334 x Female383 | 3.07 | 2.39 |
| Male334 x Female386 | 1.32 | 3.57 |
| Male334 x Female388 | 1.44 | 2.85 |
| Male334 x Female396 | 5.12 | 2.00 |
| Male334 x Female402 | 4.57 | 2.31 |
| Male334 x Female407 | 3.47 | 2.88 |
| Male334 x Female415 | 1.50 | 2.97 |
| Male334 x Female419 | 3.33 | 1.43 |
| Male334 x Female424 | 3.10 | 2.99 |
| Male334 x Female432 | 6.11 | 1.94 |
| Male334 x Female436 | 5.89 | 2.64 |
| Male334 x Female446 | 6.62 | 2.30 |
| Male334 x Female448 | 4.98 | 1.51 |
| Male334 x Female451 | 5.82 | 2.25 |
| Male334 x Female456 | 6.14 | 2.08 |
| Male334 x Female459 | 1.71 | 1.79 |
| Male334 x Female461 | 6.25 | 2.22 |
| Male334 x Female463 | 4.38 | 1.72 |
| Male334 x Female467 | 6.33 | 2.20 |
| Male334 x Female470 | 2.99 | 1.59 |
| Male334 x Female473 | 1.99 | 2.63 |
| Male334 x Female480 | 1.04 | 1.66 |
| Male334 x Female482 | 3.54 | 3.66 |
| Male334 x Female494 | 4.41 | 2.37 |
| Male334 x Female498 | 3.34 | 2.08 |
| Male334 x Female500 | 5.36 | 2.28 |
| Male335 x Female302 | 6.21 | 2.81 |
| Male335 x Female306 | 4.45 | 3.39 |
| Male335 x Female315 | 5.80 | 1.95 |
| Male335 x Female317 | 5.42 | 2.05 |
| Male335 x Female322 | 6.29 | 2.59 |
| Male335 x Female324 | 3.81 | 1.64 |
| Male335 x Female326 | 6.43 | 2.23 |
| Male335 x Female330 | 4.98 | 2.70 |
| Male335 x Female335 | 2.32 | 1.55 |
| Male335 x Female337 | 5.96 | 2.00 |
| Male335 x Female344 | 5.20 | 2.14 |
| Male335 x Female348 | 5.28 | 2.72 |
| Male335 x Female350 | 1.22 | 1.55 |
| Male335 x Female353 | 2.87 | 1.47 |
| Male335 x Female363 | 1.93 | 1.82 |
| Male335 x Female370 | 2.47 | 1.76 |
| Male335 x Female377 | 6.04 | 2.31 |
| Male335 x Female384 | 3.55 | 1.37 |
| Male335 x Female387 | 3.10 | 1.56 |
| Male335 x Female389 | 0.45 | 1.64 |
| Male335 x Female395 | 5.52 | 2.18 |
| Male335 x Female397 | 5.31 | 2.15 |
| Male335 x Female401 | 2.92 | 1.52 |
| Male335 x Female404 | 6.39 | 2.19 |
| Male335 x Female414 | 6.34 | 2.35 |
| Male335 x Female418 | 5.60 | 1.99 |
| Male335 x Female420 | 1.43 | 1.51 |
| Male335 x Female422 | 2.75 | 1.74 |
| Male335 x Female425 | 4.07 | 1.39 |
| Male335 x Female431 | 5.07 | 1.74 |
| Male335 x Female434 | 5.47 | 1.76 |
| Male335 x Female445 | 5.98 | 2.27 |
| Male335 x Female447 | 5.52 | 2.15 |
| Male335 x Female450 | 6.28 | 2.00 |
| Male335 x Female454 | 2.65 | 1.92 |
| Male335 x Female457 | 2.79 | 2.19 |
| Male335 x Female460 | 3.03 | 2.02 |
| Male335 x Female462 | 4.81 | 2.23 |
| Male335 x Female466 | 2.64 | 2.23 |
| Male335 x Female468 | 1.34 | 1.09 |
| Male335 x Female472 | 2.84 | 2.02 |
| Male335 x Female477 | 2.22 | 1.61 |
| Male335 x Female479 | 2.49 | 1.48 |
| Male335 x Female481 | 6.86 | 2.69 |
| Male335 x Female483 | 3.61 | 1.13 |
| Male335 x Female493 | 5.91 | 2.38 |
| Male335 x Female499 | 4.12 | 1.40 |
| Male336 x Female305 | 5.57 | 2.87 |
| Male336 x Female316 | 4.63 | 2.59 |
| Male336 x Female320 | 5.71 | 2.13 |
| Male336 x Female323 | 1.70 | 1.24 |
| Male336 x Female325 | 3.42 | 1.72 |
| Male336 x Female327 | 3.89 | 1.79 |
| Male336 x Female332 | 5.42 | 1.76 |
| Male336 x Female334 | 6.40 | 1.62 |
| Male336 x Female338 | 5.04 | 1.38 |
| Male336 x Female347 | 4.94 | 2.16 |
| Male336 x Female349 | 4.69 | 2.11 |
| Male336 x Female351 | 1.62 | 1.53 |
| Male336 x Female355 | 5.38 | 1.96 |
| Male336 x Female357 | 5.79 | 2.60 |
| Male336 x Female358 | 5.08 | 1.82 |
| Male336 x Female360 | 5.05 | 2.26 |
| Male336 x Female367 | 1.30 | 1.89 |
| Male336 x Female383 | 5.26 | 1.93 |
| Male336 x Female386 | 1.34 | 1.53 |
| Male336 x Female388 | 1.23 | 2.25 |
| Male336 x Female392 | 4.38 | 1.44 |
| Male336 x Female396 | 4.70 | 1.82 |
| Male336 x Female402 | 4.71 | 2.35 |
| Male336 x Female407 | 2.69 | 2.22 |
| Male336 x Female415 | 2.91 | 1.70 |
| Male336 x Female419 | 3.78 | 1.60 |
| Male336 x Female424 | 3.64 | 1.67 |
| Male336 x Female429 | 4.39 | 1.46 |
| Male336 x Female432 | 6.07 | 1.79 |
| Male336 x Female436 | 4.36 | 2.15 |
| Male336 x Female446 | 5.60 | 2.03 |
| Male336 x Female448 | 4.83 | 1.64 |
| Male336 x Female451 | 5.19 | 1.75 |
| Male336 x Female456 | 6.33 | 2.13 |
| Male336 x Female459 | 0.93 | 1.12 |
| Male336 x Female461 | 5.30 | 1.71 |
| Male336 x Female463 | 4.23 | 2.35 |
| Male336 x Female467 | 5.22 | 2.52 |
| Male336 x Female470 | 2.91 | 1.76 |
| Male336 x Female473 | 2.10 | 1.30 |
| Male336 x Female480 | 2.07 | 1.46 |
| Male336 x Female482 | 2.55 | 1.56 |
| Male336 x Female494 | 3.48 | 1.53 |
| Male336 x Female498 | 2.79 | 1.59 |
| Male336 x Female500 | 4.40 | 1.59 |
| Male337 x Female305 | 5.16 | 4.57 |
| Male337 x Female316 | 5.54 | 2.35 |
| Male337 x Female323 | 1.52 | 1.80 |
| Male337 x Female325 | 1.97 | 2.77 |
| Male337 x Female327 | 2.60 | 3.67 |
| Male337 x Female334 | 6.11 | 2.61 |
| Male337 x Female338 | 4.96 | 1.84 |
| Male337 x Female347 | 5.88 | 3.20 |
| Male337 x Female351 | 2.31 | 2.36 |
| Male337 x Female355 | 3.63 | 1.50 |
| Male337 x Female357 | 5.31 | 2.75 |
| Male337 x Female358 | 4.98 | 1.78 |
| Male337 x Female360 | 6.47 | 3.51 |
| Male337 x Female367 | 1.74 | 2.59 |
| Male337 x Female383 | 2.74 | 3.51 |
| Male337 x Female386 | 1.60 | 3.95 |
| Male337 x Female388 | 1.97 | 4.87 |
| Male337 x Female392 | 5.16 | 1.77 |
| Male337 x Female396 | 4.18 | 2.33 |
| Male337 x Female402 | 5.11 | 2.51 |
| Male337 x Female407 | 1.81 | 3.62 |
| Male337 x Female415 | 2.07 | 4.71 |
| Male337 x Female419 | 2.65 | 1.65 |
| Male337 x Female424 | 2.20 | 2.17 |
| Male337 x Female429 | 4.48 | 2.30 |
| Male337 x Female432 | 5.19 | 1.96 |
| Male337 x Female436 | 4.35 | 2.93 |
| Male337 x Female446 | 5.19 | 3.80 |
| Male337 x Female448 | 2.59 | 1.29 |
| Male337 x Female451 | 4.59 | 3.04 |
| Male337 x Female456 | 5.20 | 1.53 |
| Male337 x Female459 | 1.52 | 1.39 |
| Male337 x Female461 | 4.37 | 2.98 |
| Male337 x Female463 | 3.38 | 2.92 |
| Male337 x Female467 | 5.48 | 2.39 |
| Male337 x Female470 | 2.07 | 2.32 |
| Male337 x Female473 | 1.56 | 3.75 |
| Male337 x Female480 | 1.75 | 1.46 |
| Male337 x Female482 | 1.90 | 3.12 |
| Male337 x Female489 | 4.15 | 2.03 |
| Male337 x Female494 | 3.62 | 1.86 |
| Male337 x Female498 | 2.29 | 2.96 |
| Male337 x Female500 | 4.37 | 2.68 |
| Male338 x Female302 | 4.59 | 2.37 |
| Male338 x Female306 | 2.39 | 2.18 |
| Male338 x Female315 | 2.90 | 2.01 |
| Male338 x Female317 | 4.30 | 1.50 |
| Male338 x Female322 | 3.97 | 1.88 |
| Male338 x Female324 | 2.49 | 1.51 |
| Male338 x Female330 | 1.94 | 1.53 |
| Male338 x Female335 | 2.26 | 1.51 |
| Male338 x Female337 | 3.26 | 2.30 |
| Male338 x Female344 | 3.00 | 1.60 |
| Male338 x Female348 | 2.23 | 1.43 |
| Male338 x Female363 | 2.47 | 1.81 |
| Male338 x Female370 | 2.57 | 2.17 |
| Male338 x Female377 | 3.64 | 1.93 |
| Male338 x Female384 | 1.91 | 1.29 |
| Male338 x Female387 | 2.72 | 1.80 |
| Male338 x Female389 | 2.33 | 2.43 |
| Male338 x Female395 | 2.22 | 1.41 |
| Male338 x Female397 | 2.73 | 1.37 |
| Male338 x Female404 | 4.47 | 1.76 |
| Male338 x Female416 | 2.08 | 1.59 |
| Male338 x Female418 | 3.00 | 1.50 |
| Male338 x Female420 | 1.39 | 1.37 |
| Male338 x Female422 | 2.05 | 1.89 |
| Male338 x Female425 | 1.99 | 1.98 |
| Male338 x Female445 | 5.44 | 1.98 |
| Male338 x Female450 | 3.34 | 1.62 |
| Male338 x Female460 | 2.16 | 1.90 |
| Male338 x Female462 | 1.94 | 2.07 |
| Male338 x Female466 | 2.54 | 3.36 |
| Male338 x Female472 | 3.37 | 2.02 |
| Male338 x Female477 | 1.29 | 1.31 |
| Male338 x Female481 | 3.08 | 2.18 |
| Male338 x Female483 | 1.93 | 1.39 |
| Male338 x Female493 | 2.77 | 2.46 |
| Male338 x Female499 | 3.17 | 1.45 |
| Male339 x Female305 | 6.38 | 3.13 |
| Male339 x Female316 | 5.66 | 2.58 |
| Male339 x Female320 | 6.01 | 2.94 |
| Male339 x Female323 | 1.79 | 1.75 |
| Male339 x Female325 | 3.26 | 2.55 |
| Male339 x Female327 | 1.92 | 3.36 |
| Male339 x Female332 | 5.61 | 2.60 |
| Male339 x Female334 | 5.50 | 2.82 |
| Male339 x Female336 | 5.07 | 2.35 |
| Male339 x Female338 | 5.90 | 1.71 |
| Male339 x Female347 | 5.53 | 3.56 |
| Male339 x Female349 | 4.71 | 2.90 |
| Male339 x Female351 | 1.74 | 3.62 |
| Male339 x Female355 | 5.40 | 1.91 |
| Male339 x Female357 | 3.82 | 2.02 |
| Male339 x Female358 | 5.68 | 2.07 |
| Male339 x Female360 | 6.60 | 3.00 |
| Male339 x Female367 | 2.38 | 2.90 |
| Male339 x Female383 | 2.58 | 3.40 |
| Male339 x Female386 | 1.19 | 4.47 |
| Male339 x Female388 | 1.38 | 4.54 |
| Male339 x Female392 | 4.42 | 1.81 |
| Male339 x Female396 | 5.79 | 2.25 |
| Male339 x Female402 | 4.31 | 2.75 |
| Male339 x Female407 | 1.97 | 2.78 |
| Male339 x Female415 | 2.38 | 5.84 |
| Male339 x Female417 | 5.27 | 4.30 |
| Male339 x Female419 | 3.97 | 2.13 |
| Male339 x Female424 | 2.68 | 3.07 |
| Male339 x Female429 | 4.54 | 2.10 |
| Male339 x Female432 | 5.91 | 1.44 |
| Male339 x Female436 | 6.28 | 2.12 |
| Male339 x Female446 | 5.63 | 3.52 |
| Male339 x Female448 | 2.42 | 1.40 |
| Male339 x Female451 | 5.90 | 3.22 |
| Male339 x Female456 | 5.81 | 1.93 |
| Male339 x Female459 | 1.58 | 1.44 |
| Male339 x Female461 | 4.40 | 3.11 |
| Male339 x Female463 | 3.78 | 2.58 |
| Male339 x Female467 | 6.16 | 2.55 |
| Male339 x Female470 | 1.48 | 2.70 |
| Male339 x Female473 | 1.29 | 4.58 |
| Male339 x Female480 | 1.35 | 1.31 |
| Male339 x Female482 | 2.47 | 3.38 |
| Male339 x Female489 | 6.24 | 2.07 |
| Male339 x Female494 | 3.14 | 2.63 |
| Male339 x Female498 | 1.83 | 3.22 |
| Male339 x Female500 | 4.37 | 2.73 |
| Male340 x Female305 | 5.45 | 2.72 |
| Male340 x Female314 | 1.41 | 1.27 |
| Male340 x Female316 | 4.46 | 1.61 |
| Male340 x Female320 | 5.45 | 1.97 |
| Male340 x Female323 | 2.85 | 2.41 |
| Male340 x Female325 | 2.77 | 1.58 |
| Male340 x Female327 | 2.65 | 4.11 |
| Male340 x Female332 | 5.72 | 4.48 |
| Male340 x Female334 | 5.55 | 2.46 |
| Male340 x Female336 | 4.03 | 3.06 |
| Male340 x Female338 | 3.76 | 2.15 |
| Male340 x Female347 | 5.42 | 2.70 |
| Male340 x Female349 | 3.73 | 3.06 |
| Male340 x Female351 | 1.75 | 3.46 |
| Male340 x Female355 | 2.86 | 2.01 |
| Male340 x Female357 | 5.19 | 2.61 |
| Male340 x Female358 | 4.08 | 1.62 |
| Male340 x Female360 | 5.40 | 2.90 |
| Male340 x Female367 | 1.81 | 2.18 |
| Male340 x Female383 | 3.28 | 3.75 |
| Male340 x Female386 | 2.35 | 3.81 |
| Male340 x Female388 | 2.18 | 5.42 |
| Male340 x Female392 | 5.42 | 1.60 |
| Male340 x Female396 | 5.00 | 1.49 |
| Male340 x Female402 | 2.77 | 2.83 |
| Male340 x Female407 | 2.72 | 3.18 |
| Male340 x Female415 | 3.13 | 4.78 |
| Male340 x Female417 | 4.90 | 2.74 |
| Male340 x Female419 | 2.27 | 1.71 |
| Male340 x Female421 | 4.17 | 3.63 |
| Male340 x Female424 | 2.48 | 1.88 |
| Male340 x Female429 | 3.81 | 2.79 |
| Male340 x Female432 | 5.20 | 1.48 |
| Male340 x Female436 | 3.57 | 2.28 |
| Male340 x Female446 | 4.35 | 3.58 |
| Male340 x Female448 | 3.48 | 1.41 |
| Male340 x Female451 | 4.40 | 3.17 |
| Male340 x Female456 | 4.75 | 1.46 |
| Male340 x Female459 | 2.01 | 1.66 |
| Male340 x Female461 | 4.48 | 3.16 |
| Male340 x Female463 | 4.83 | 2.58 |
| Male340 x Female467 | 4.02 | 2.31 |
| Male340 x Female470 | 2.18 | 2.13 |
| Male340 x Female473 | 2.28 | 3.05 |
| Male340 x Female480 | 1.40 | 1.45 |
| Male340 x Female482 | 2.71 | 3.39 |
| Male340 x Female489 | 4.51 | 2.67 |
| Male340 x Female494 | 2.51 | 2.95 |
| Male340 x Female498 | 1.89 | 2.09 |
| Male340 x Female500 | 5.05 | 3.21 |

**Suppl. Table 2:** Loci significantly associated with leaf rust resistance, their chromosomal (Chrom.) position (Pos.), the additive (Add.) and dominance (Dom.) effects, and the adjusted (Adj.) R².

| **Marker** | **Type** | **Chrom.** | **Pos. (cM)** | **Effect** | **adj. R^2^ (%)** |
| --- | --- | --- | --- | --- | --- |
| TA022870-0883 | Add | 1D | 63.16 | -0.91 | 6.98 |
| BobWhite_c12911_788 | Add | 2B | 136.34 | -0.33 | 1.64 |
| wsnp_CAP11_c3226_1588070 | Add | 2B | 136.56 | -0.54 | 5.92 |
| Kukri_c43464_89 | Add | 3D | 169.86 | -1.65 | 14.87 |
| RAC875_c31922_138 | Add/Dom | 3D | 169.86 | -1.66 | 14.77 |
| D_GBUVHFX02FQKHM_48 | Add/Dom | 3D | 170.19 | -1.72 | 16.51 |
| Excalibur_c51312_218 | Add/Dom | 3D | 170.19 | -1.66 | 14.77 |
| Kukri_c14943_753 | Add/Dom | 3D | 170.39 | -1.66 | 14.77 |
| Kukri_c4230_398 | Add/Dom | 3D | 170.39 | -1.73 | 15.55 |
| RAC875_c61950_1644 | Add/Dom | 3D | 170.39 | -1.66 | 14.77 |
| Kukri_c16352_687 | Add | 3D | 174.14 | -1.47 | 11.87 |
| BobWhite_c2661_155 | Add | 3D | 175.39 | -1.47 | 11.87 |
| Ra_c6639_426 | Add/Dom | 3D | 175.39 | -1.65 | 16.76 |
| Ra_c72650_1176 | Add/Dom | 3D | 175.39 | -1.73 | 15.55 |
| TA015516-0532 | Add | 3D | 175.39 | -1.48 | 12.10 |
| TA024439-0060 | Add | 3D | 175.39 | -1.48 | 13.01 |
| wsnp_Ex_c7451_12757458 | Add | 3D | 177.21 | -1.05 | 8.13 |
| CAP12_rep_c3953_177 | Add/Dom | 3D | 177.39 | -1.66 | 14.77 |
| Kukri_c23354_183 | Add/Dom | 3D | 177.39 | -1.66 | 13.57 |
| RAC875_c61950_377 | Add/Dom | 3D | 177.39 | -1.68 | 13.83 |
| wsnp_Ra_c7174_12417331 | Add/Dom | 3D | 177.39 | -1.66 | 14.77 |
| BS00033229_51 | Add | 3D | 178.73 | -1.04 | 8.09 |
| BobWhite_rep_c52911_146 | Add | 3D | 179.88 | -1.05 | 8.17 |
| Excalibur_c42667_427 | Add | 3D | 181.26 | -0.66 | 4.07 |
| IAAV686 | Add | 3D | 181.26 | -1.05 | 8.03 |
| IAAV7013 | Add | 3D | 181.26 | -1.06 | 8.28 |
| wsnp_Ex_c14027_21925404 | Add/Dom | 3D | 181.26 | -1.66 | 14.77 |
| Kukri_c20012_1425 | Dom | 4A | 103.66 | -0.68 | 9.77 |
| BS00072157_51 | Dom | 4A | 110.84 | -0.71 | 10.41 |
| BS00039148_51 | Dom | 4A | 113.39 | -0.76 | 12.06 |
| wsnp_Ex_c2352_4405961 | Dom | 4A | 113.39 | -0.69 | 9.79 |
| Kukri_c48943_1149 | Dom | 4A | 113.55 | -0.77 | 12.10 |
| RAC875_c95150_286 | Dom | 4A | 113.55 | -0.74 | 11.89 |
| IAAV1383 | Dom | 4A | 117.85 | -0.78 | 12.68 |
| BobWhite_s66966_118 | Add | 4A | 126.68 | -0.70 | 10.88 |
| BS00084703_51 | Add | 4A | 126.68 | -0.72 | 11.15 |
| wsnp_Ex_c4331_7808746 | Add | 4A | 126.68 | -0.71 | 11.19 |
| wsnp_Ex_c6094_10663424 | Add | 4A | 126.68 | -0.70 | 10.84 |
| GENE-0689_30 | Add/Dom | 4A | 136.51 | -1.12 | 21.86 |
| Tdurum_contig75819_1220 | Dom | 4A | 137.31 | 0.46 | 2.19 |
| BobWhite_c11327_185 | Add/Dom | 4A | 137.70 | -1.13 | 22.18 |
| RFL_Contig3841_2433 | Dom | 4A | 139.00 | -0.71 | 10.77 |
| Tdurum_contig45738_670 | Add/Dom | 4A | 139.00 | -1.14 | 22.05 |
| Excalibur_c33542_113 | Add | 4A | 139.14 | -0.76 | 9.80 |
| Excalibur_rep_c112888_602 | Add/Dom | 4A | 139.14 | -1.14 | 22.25 |
| RAC875_rep_c69632_65 | Add | 4A | 139.14 | -0.76 | 12.15 |
| BobWhite_c20306_88 | Add/Dom | 4A | 139.17 | -1.14 | 22.36 |
| BS00110021_51 | Add/Dom | 4A | 139.17 | -0.91 | 16.28 |
| BobWhite_c47168_289 | Add | 4A | 149.75 | -0.72 | 11.78 |
| BobWhite_c47168_598 | Add | 4A | 149.75 | -0.73 | 12.21 |
| RFL_Contig2531_1872 | Add/Dom | 4A | 149.75 | -1.14 | 22.24 |
| Tdurum_contig46583_2203 | Add/Dom | 4A | 151.07 | -0.88 | 15.24 |
| Tdurum_contig47476_528 | Add/Dom | 4A | 152.21 | -1.09 | 20.64 |
| Tdurum_contig47476_495 | Add/Dom | 4A | 152.31 | -1.09 | 20.70 |
| Excalibur_c46904_84 | Add/Dom | 4A | 153.01 | -1.02 | 18.85 |
| IACX5724 | Add/Dom | 4A | 153.01 | -1.02 | 19.05 |
| RAC875_c4800_255 | Add/Dom | 4A | 153.01 | -1.02 | 18.94 |
| Tdurum_contig46583_1275 | Add/Dom | 4A | 153.64 | -0.97 | 18.61 |
| BobWhite_c25163_178 | Add/Dom | 4A | 161.19 | -0.93 | 16.35 |
| Excalibur_rep_c66939_849 | Add/Dom | 4A | 161.19 | -0.97 | 18.27 |
| wsnp_Ex_c33012_41567026 | Add/Dom | 4A | 161.19 | -0.99 | 18.89 |
| Kukri_rep_c85536_598 | Add/Dom | 4A | 161.69 | -1.06 | 19.68 |
| Tdurum_contig54776_1396 | Add | 4A | 165.81 | -0.85 | 13.69 |
| wsnp_Ex_c10955_17794520 | Add/Dom | 4A | 165.81 | -0.91 | 16.54 |
| wsnp_Ex_c5072_9006966 | Add/Dom | 4A | 165.81 | -0.86 | 15.07 |
| wsnp_Ex_c8976_14964359 | Add/Dom | 4A | 165.81 | -0.92 | 16.30 |
| wsnp_Ex_rep_c67099_65575038 | Add/Dom | 4A | 165.81 | -0.87 | 15.60 |
| wsnp_Ku_c9746_16265584 | Add/Dom | 4A | 165.81 | -0.91 | 15.84 |
| tplb0057e06_1607 | Dom | 6A | 27.19 | -0.33 | 1.57 |
| BobWhite_c25234_418 | Add | 7A | 27.31 | -0.77 | 12.01 |
| tplb0057f21_914 | Add/Dom | 7D | 7.73 | -0.86 | 14.48 |
| IACX7717 | Add | Unmapped | NA | -0.23 | 0.84 |
| Jagger_rep_c10298_150 | Add/Dom | Unmapped | NA | -1.12 | 21.52 |
| RAC875_c195_499 | Dom | Unmapped | NA | -0.76 | 13.93 |
| Excalibur_c22830_1989 | Add/Dom | Unmapped | NA | -1.08 | 20.99 |
| Excalibur_rep_c69170_438 | Add/Dom | Unmapped | NA | -0.99 | 17.81 |
| JD_c7714_954 | Add/Dom | Unmapped | NA | -1.66 | 14.77 |

**Suppl. Table 3:** Loci significantly associated with stripe rust resistance, their chromosomal (Chrom.) position (Pos.), the additive (Add.) and dominance (Dom.) effects, and the adjusted (Adj.) R².

| **Marker** | **Type** | **Chrom.** | **Pos. (cM)** | **Effect** | **adj. R^2^ (%)** |
| --- | --- | --- | --- | --- | --- |
| RAC875_c1226_652 | Add | 2B | 78.22 | -0.13 | 0.19 |
| Excalibur_c20459_1081 | Add | 6A | 121.65 | -0.34 | 2.49 |
| BobWhite_c40602_313 | Add | 6A | 122.84 | -0.24 | 0.89 |
| Tdurum_contig29607_413 | Add | 6A | 122.84 | 0.43 | 3.09 |
| Kukri_c1468_1609 | Add | 6A | 128.42 | -0.29 | 1.28 |
| RFL_Contig5262_1500 | Add | 6A | 128.42 | -0.29 | 1.28 |
| wsnp_RFL_Contig4456_5258284 | Add | 6A | 129.73 | 0.39 | 2.82 |
| Excalibur_rep_c69054_795 | Add | 6A | 130.57 | -0.31 | 2.09 |
| BS00040814_51 | Add | 6A | 133.29 | 0.35 | 2.86 |
| Excalibur_rep_c67100_1541 | Add | 6A | 133.29 | -0.31 | 2.12 |
| Jagger_c5046_63 | Add | 6A | 133.29 | 0.41 | 3.19 |
| RAC875_c8721_212 | Add | 6A | 133.29 | 0.42 | 3.3 |
| wsnp_Ku_rep_c113718_96236830 | Add | 7A | 116.01 | 0.13 | 0.4 |
| CAP8_c2210_103 | Add | Unmapped | NA | -0.29 | 1.28 |
| TA005377.1076 | Add | Unmapped | NA | -0.27 | 0.94 |

**Suppl. Table 4:** Degree of dominance for markers significantly associated to leaf rust and stripe rust resistance overlapping between this current study and a previous study of Beukert et al. (2020).

| **Marker** | **Disease** | **Current Study** | | | **Previous study** | | |
| --- | --- | --- | --- | --- | --- | --- | --- |
|  |  | **Type** | **Add. Effect** | **Dom. Effect** | **Type** | **Add. Effect** | **Dom. Effect** |
| BobWhite_c11327_185 | Leaf rust | Add/Dom | 70.65 | 109.45 | Add/Dom | 0.17 | -0.23 |
| BobWhite_c20306_88 | Leaf rust | Add/Dom | 75.84 | 113.87 | Add/Dom | -0.17 | -0.23 |
| BobWhite_c25163_178 | Leaf rust | Add/Dom | 22.33 | 76.43 | Add | 0.17 | -0.18 |
| BobWhite_c25234_418 | Leaf rust | Add | 24.36 | 6.82 | Add | 0.16 | -0.17 |
| BobWhite_c47168_289 | Leaf rust | Add | 32.39 | 6.58 | Add | 0.18 | -0.15 |
| BobWhite_c47168_598 | Leaf rust | Add | 37.14 | 6.60 | Add/Dom | -0.19 | -0.14 |
| BobWhite_s66966_118 | Leaf rust | Add | 24.42 | 10.13 | Add | 0.17 | -0.11 |
| BS00039148_51 | Leaf rust | Dom | 6.34 | 50.85 | Add | -0.17 | -0.21 |
| BS00072157_51 | Leaf rust | Dom | 3.15 | 38.82 | Add | 0.17 | -0.19 |
| BS00084703_51 | Leaf rust | Add | 21.22 | 11.96 | Add | 0.17 | -0.13 |
| BS00110021_51 | Leaf rust | Add/Dom | 48.85 | 35.27 | Add/Dom | 0.17 | -0.21 |
| Excalibur_c33542_113 | Leaf rust | Add | 23.12 | 9.18 | Add | 0.16 | -0.16 |
| Excalibur_c46904_84 | Leaf rust | Add/Dom | 39.61 | 83.36 | Add | 0.17 | -0.2 |
| Excalibur_rep_c112888_602 | Leaf rust | Add/Dom | 70.30 | 116.25 | Add/Dom | -0.16 | -0.24 |
| Excalibur_rep_c66939_849 | Leaf rust | Add/Dom | 46.25 | 45.14 | Add | -0.18 | -0.19 |
| GENE-0689_30 | Leaf rust | Add/Dom | 57.38 | 107.54 | Add | -0.16 | -0.21 |
| IAAV1383 | Leaf rust | Dom | 10.95 | 42.35 | Add | 0.19 | -0.18 |
| IACX5724 | Leaf rust | Add/Dom | 45.44 | 83.00 | Add | 0.17 | -0.2 |
| Jagger_rep_c10298_150 | Leaf rust | Add/Dom | 60.65 | 109.76 | Add/Dom | -0.17 | -0.22 |
| Kukri_c20012_1425 | Leaf rust | Dom | 4.16 | 33.27 | Add | -0.17 | -0.22 |
| Kukri_c4230_398 | Leaf rust | Add/Dom | 79.84 | 23.83 | Dom | -0.18 | -0.09 |
| Kukri_c48943_1149 | Leaf rust | Dom | 4.28 | 59.26 | Add | -0.17 | -0.18 |
| Kukri_rep_c85536_598 | Leaf rust | Add/Dom | 48.77 | 90.01 | Add | 0.17 | -0.23 |
| Ra_c6639_426 | Leaf rust | Add/Dom | 71.01 | 35.76 | Dom | 0.18 | -0.15 |
| Ra_c72650_1176 | Leaf rust | Add/Dom | 79.84 | 23.83 | Dom | -0.18 | -0.1 |
| RAC875_c4800_255 | Leaf rust | Add/Dom | 39.87 | 82.34 | Add | -0.17 | -0.2 |
| RAC875_c95150_286 | Leaf rust | Dom | 6.57 | 46.99 | Add | 0.18 | -0.14 |
| RAC875_rep_c69632_65 | Leaf rust | Add | 26.38 | 12.18 | Add | 0.16 | -0.16 |
| RFL_Contig2531_1872 | Leaf rust | Add/Dom | 73.40 | 115.58 | Add/Dom | 0.17 | -0.25 |
| RFL_Contig3841_2433 | Leaf rust | Dom | 18.78 | 21.01 | Add | -0.18 | -0.13 |
| Tdurum_contig45738_670 | Leaf rust | Add/Dom | 59.79 | 108.45 | Add/Dom | 0.16 | -0.25 |
| Tdurum_contig46583_1275 | Leaf rust | Add/Dom | 48.29 | 47.74 | Add | -0.17 | -0.19 |
| Tdurum_contig46583_2203 | Leaf rust | Add/Dom | 24.56 | 44.74 | Add | -0.18 | -0.18 |
| Tdurum_contig47476_495 | Leaf rust | Add/Dom | 34.47 | 110.81 | Add | 0.17 | -0.22 |
| Tdurum_contig47476_528 | Leaf rust | Add/Dom | 33.69 | 109.45 | Add | -0.17 | -0.22 |
| Tdurum_contig54776_1396 | Leaf rust | Add | 29.62 | 13.50 | Add | -0.17 | -0.15 |
| tplb0057f21_914 | Leaf rust | Add/Dom | 21.82 | 43.57 | Add | 0.17 | -0.21 |
| wsnp_Ex_c10955_17794520 | Leaf rust | Add/Dom | 29.34 | 41.34 | Add | 0.17 | -0.13 |
| wsnp_Ex_c2352_4405961 | Leaf rust | Dom | 8.89 | 45.89 | Add | -0.17 | -0.17 |
| wsnp_Ex_c33012_41567026 | Leaf rust | Add/Dom | 37.27 | 43.72 | Add | -0.16 | -0.14 |
| wsnp_Ex_c4331_7808746 | Leaf rust | Add | 21.73 | 11.55 | Add | -0.17 | -0.12 |
| wsnp_Ex_c5072_9006966 | Leaf rust | Add/Dom | 33.06 | 52.56 | Add | 0.18 | -0.14 |
| wsnp_Ex_c8976_14964359 | Leaf rust | Add/Dom | 36.42 | 73.71 | Add | 0.17 | -0.15 |
| wsnp_Ex_rep_c67099_65575038 | Leaf rust | Add/Dom | 35.24 | 45.56 | Add | 0.18 | -0.13 |
| wsnp_Ku_c9746_16265584 | Leaf rust | Add/Dom | 29.81 | 26.14 | Add | -0.17 | -0.14 |
| RAC875_c1226_652 | Stripe rust | Add | 31.83 | 10.14 | Dom | -0.28 | -0.19 |
| Jagger_c5046_63 | Stripe rust | Add | 35.28 | 13.48 | Dom | -0.17 | -0.08 |
| wsnp_RFL_Contig4456_5258284 | Stripe rust | Add | 34.87 | 14.47 | Dom | 0.18 | -0.08 |
| RAC875_c8721_212 | Stripe rust | Add | 43.02 | 17.68 | Dom | -0.17 | -0.09 |
| Tdurum_contig29607_413 | Stripe rust | Add | 34.93 | 16.31 | Dom | -0.19 | -0.04 |
| BS00040814_51 | Stripe rust | Add | 27.65 | 11.29 | Dom | -0.08 | -0.12 |

**Suppl. Table 5:** Comparison of markers significantly associated to leaf rust and stripe rust resistance in the previous study of Beukert et al. (2020) and the current study giving their explained phenotypic variance (Adj. R2), while green color represents a p-value of <0.1 and <0.05 for previous and current study, respectively. Red color symbolizes the missed identification due to a higher p-value.

| **Marker** | **Disease** | **Previous study** | | **Current Study** | |
| --- | --- | --- | --- | --- | --- |
|  |  | **Type** | **Adj. R**^2^ (%) | **Type** | **Adj. R**^2^ (%) |
| BobWhite_c11327_185 | Leaf rust | Add/Dom | 11,83 | Add/Dom | 22,18 |
| BobWhite_c12911_788 | Leaf rust |  |  | Add | 1,64 |
| BobWhite_c14495_230 | Leaf rust | Add | 6,73 |  |  |
| BobWhite_c20306_88 | Leaf rust | Add/Dom | 11,87 | Add/Dom | 22,36 |
| BobWhite_c25163_178 | Leaf rust | Add | 8,95 | Add/Dom | 16,35 |
| BobWhite_c25234_418 | Leaf rust | Add | 8,51 | Add | 12,01 |
| BobWhite_c2661_155 | Leaf rust |  |  | Add | 11,87 |
| BobWhite_c47168_289 | Leaf rust | Add | 8,45 | Add | 11,78 |
| BobWhite_c47168_598 | Leaf rust | Add/Dom | 8,95 | Add | 12,21 |
| BobWhite_c8680_918 | Leaf rust | Add | 6,61 |  |  |
| BobWhite_rep_c52911_146 | Leaf rust |  |  | Add | 8,17 |
| BobWhite_s66966_118 | Leaf rust | Add | 6,38 |  |  |
| BS00033229_51 | Leaf rust |  |  | Add | 8,09 |
| BS00039148_51 | Leaf rust | Add | 10,66 |  |  |
| BS00048067_51 | Leaf rust | Add | 7,21 |  |  |
| BS00059454_51 | Leaf rust | Add | 6,16 |  |  |
| BS00065030_51 | Leaf rust | Add | 5,53 |  |  |
| BS00072157_51 | Leaf rust | Add | 9,99 |  |  |
| BS00084703_51 | Leaf rust | Add | 7,24 |  |  |
| BS00091561_51 | Leaf rust | Add | 6,61 |  |  |
| BS00099983_51 | Leaf rust | Add | 6,76 |  |  |
| BS00110021_51 | Leaf rust | Add/Dom | 10,44 |  |  |
| CAP12_rep_c3953_177 | Leaf rust |  |  | Add/Dom | 14,77 |
| CAP7_c254_486 | Leaf rust | Add | 7,11 |  |  |
| D_GBUVHFX02FQKHM_48 | Leaf rust |  |  | Add/Dom | 16,51 |
| Ex_c101546_376 | Leaf rust | Add | 7,57 |  |  |
| Excalibur_c13811_1086 | Leaf rust | Add | 6,07 |  |  |
| Excalibur_c22830_1989 | Leaf rust |  |  | Add/Dom | 20,99 |
| Excalibur_c33542_113 | Leaf rust | Add | 8,18 |  |  |
| Excalibur_c42667_427 | Leaf rust |  |  | Add | 4,07 |
| Excalibur_c46904_84 | Leaf rust | Add | 10,35 |  |  |
| Excalibur_c51312_218 | Leaf rust |  |  | Add/Dom | 14,77 |
| Excalibur_rep_c112888_602 | Leaf rust | Add/Dom | 11,22 |  |  |
| Excalibur_rep_c66939_849 | Leaf rust | Add | 10,32 |  |  |
| Excalibur_rep_c69170_438 | Leaf rust |  |  | Add/Dom | 17,81 |
| GENE-0689_30 | Leaf rust | Add | 10,34 |  |  |
| IAAV1383 | Leaf rust | Add | 10,43 |  |  |
| IAAV686 | Leaf rust |  |  | Add | 8,03 |
| IAAV7013 | Leaf rust |  |  | Add | 8,28 |
| IACX5724 | Leaf rust | Add | 10,15 |  |  |
| IACX7717 | Leaf rust |  |  | Add | 0,84 |
| IACX8322 | Leaf rust | Add | 7,22 |  |  |
| Jagger_rep_c10298_150 | Leaf rust | Add/Dom | 11,08 |  |  |
| JD_c7714_954 | Leaf rust |  |  | Add/Dom | 14,77 |
| Kukri_c14943_753 | Leaf rust |  |  | Add/Dom | 14,77 |
| Kukri_c16352_687 | Leaf rust |  |  | Add | 11,87 |
| Kukri_c20012_1425 | Leaf rust | Add | 11,04 |  |  |
| Kukri_c23354_183 | Leaf rust |  |  | Add/Dom | 13,57 |
| Kukri_c27648_350 | Leaf rust | Add | 7,15 |  |  |
| Kukri_c4230_398 | Leaf rust | Dom | 2,94 |  |  |
| Kukri_c43464_89 | Leaf rust |  |  | Add | 14,87 |
| Kukri_c48943_1149 | Leaf rust | Add | 9,29 |  |  |
| Kukri_rep_c85536_598 | Leaf rust | Add | 11,27 |  |  |
| Ra_c6639_426 | Leaf rust | Dom | 4,45 |  |  |
| Ra_c72650_1176 | Leaf rust | Dom | 3,13 |  |  |
| RAC875_c195_499 | Leaf rust |  |  | Dom | 13,93 |
| RAC875_c31922_138 | Leaf rust |  |  | Add/Dom | 14,77 |
| RAC875_c4800_255 | Leaf rust | Add | 10,03 |  |  |
| RAC875_c61950_1644 | Leaf rust |  |  | Add/Dom | 14,77 |
| RAC875_c61950_377 | Leaf rust |  |  | Add/Dom | 13,83 |
| RAC875_c67909_226 | Leaf rust | Add | 6,11 |  |  |
| RAC875_c88582_131 | Leaf rust | Add | 7,52 |  |  |
| RAC875_c95150_286 | Leaf rust | Add | 8,29 |  |  |
| RAC875_rep_c104791_336 | Leaf rust | Add | 6,67 |  |  |
| RAC875_rep_c106400_276 | Leaf rust | Add | 4,05 |  |  |
| RAC875_rep_c112008_519 | Leaf rust | Add | 4,25 |  |  |
| RAC875_rep_c69632_65 | Leaf rust | Add | 8,00 |  |  |
| RFL_Contig2531_1872 | Leaf rust | Add/Dom | 12,31 |  |  |
| RFL_Contig2531_987 | Leaf rust | Add | 4,85 |  |  |
| RFL_Contig3841_1986 | Leaf rust | Add | 7,62 |  |  |
| RFL_Contig3841_2433 | Leaf rust | Add | 7,96 |  |  |
| RFL_Contig3841_2595 | Leaf rust | Add | 7,62 |  |  |
| RFL_Contig4336_184 | Leaf rust | Add | 7,01 |  |  |
| TA004646-0293 | Leaf rust | Add | 6,08 |  |  |
| TA015516-0532 | Leaf rust |  |  | Add | 12,10 |
| TA022870-0883 | Leaf rust |  |  | Add | 6,98 |
| TA024439-0060 | Leaf rust |  |  | Add | 13,01 |
| Tdurum_contig41127_265 | Leaf rust | Add | 8,20 |  |  |
| Tdurum_contig45738_670 | Leaf rust | Add/Dom | 12,00 |  |  |
| Tdurum_contig46583_1275 | Leaf rust | Add | 9,82 |  |  |
| Tdurum_contig46583_2203 | Leaf rust | Add | 10,01 |  |  |
| Tdurum_contig47476_495 | Leaf rust | Add | 11,35 |  |  |
| Tdurum_contig47476_528 | Leaf rust | Add | 11,00 |  |  |
| Tdurum_contig54776_1396 | Leaf rust | Add | 8,41 |  |  |
| Tdurum_contig75819_1220 | Leaf rust |  |  | Dom | 2,19 |
| Tdurum_contig7992_605 | Leaf rust | Add | 6,64 |  |  |
| Tdurum_contig93100_149 | Leaf rust | Add | 6,52 |  |  |
| tplb0057e06_1607 | Leaf rust |  |  | Dom | 1,57 |
| tplb0057f21_914 | Leaf rust | Add | 10,30 |  |  |
| wsnp_CAP11_c3226_1588070 | Leaf rust |  |  | Add | 5,92 |
| wsnp_CAP7_c254_138937 | Leaf rust | Add | 7,44 |  |  |
| wsnp_Ex_c10955_17794520 | Leaf rust | Add | 7,65 |  |  |
| wsnp_Ex_c14027_21925404 | Leaf rust |  |  | Add/Dom | 14,77 |
| wsnp_Ex_c2352_4405961 | Leaf rust | Add | 8,56 |  |  |
| wsnp_Ex_c33012_41567026 | Leaf rust | Add | 7,26 |  |  |
| wsnp_Ex_c4331_7808746 | Leaf rust | Add | 6,96 |  |  |
| wsnp_Ex_c5072_9006966 | Leaf rust | Add | 8,42 |  |  |
| wsnp_Ex_c6094_10663424 | Leaf rust |  |  | Add | 10,84 |
| wsnp_Ex_c7451_12757458 | Leaf rust |  |  | Add | 8,13 |
| wsnp_Ex_c8976_14964359 | Leaf rust | Add | 8,01 |  |  |
| wsnp_Ex_rep_c67099_65575038 | Leaf rust | Add | 8,01 |  |  |
| wsnp_Ku_c20783_30448706 | Leaf rust | Add | 7,64 |  |  |
| wsnp_Ku_c9746_16265584 | Leaf rust | Add | 7,99 |  |  |
| wsnp_Ra_c7174_12417331 | Leaf rust |  |  | Add/Dom | 14,77 |
| BobWhite_c10215_242 | Stripe rust | Dom | 5,68 |  |  |
| BobWhite_c12426_84 | Stripe rust | Add/Dom | 8,15 |  |  |
| BobWhite_c13373_250 | Stripe rust | Dom | 10,78 |  |  |
| BobWhite_c16735_131 | Stripe rust | Dom | 5,16 |  |  |
| BobWhite_c17047_268 | Stripe rust | Add | 8,69 |  |  |
| BobWhite_c40602_313 | Stripe rust |  |  | Add | 0,89 |
| BS00022532_51 | Stripe rust | Add/Dom | 6,45 |  |  |
| BS00040814_51 | Stripe rust |  |  | Add | 2,86 |
| BS00040814_51 | Stripe rust | Dom | 1,37 |  |  |
| BS00067382_51 | Stripe rust | Add/Dom | 7,03 |  |  |
| BS00068050_51 | Stripe rust | Dom | 11,01 |  |  |
| BS00076693_51 | Stripe rust | Add | 5,74 |  |  |
| BS00093111_51 | Stripe rust | Add/Dom | 7,53 |  |  |
| BS00093990_51 | Stripe rust | Add/Dom | 8,56 |  |  |
| CAP12_c259_307 | Stripe rust | Add | 4,79 |  |  |
| CAP8_c2210_103 | Stripe rust |  |  | Add | 1,28 |
| D_contig01272_220 | Stripe rust | Add/Dom | 6,98 |  |  |
| Excalibur_c18324_390 | Stripe rust | Add/Dom | 8,15 |  |  |
| Excalibur_c20459_1081 | Stripe rust |  |  | Add | 2,49 |
| Excalibur_c21663_145 | Stripe rust | Add/Dom | 11,77 |  |  |
| Excalibur_c25599_358 | Stripe rust | Add/Dom | 8,21 |  |  |
| Excalibur_c62893_629 | Stripe rust | Add/Dom | 6,08 |  |  |
| Excalibur_rep_c67100_1541 | Stripe rust |  |  | Add | 2,12 |
| Excalibur_rep_c69054_795 | Stripe rust |  |  | Add | 2,09 |
| GENE-4021_496 | Stripe rust | Dom | 2,56 |  |  |
| IAAV1743 | Stripe rust | Dom | 5,54 |  |  |
| IAAV5607 | Stripe rust | Dom | 1,55 |  |  |
| IAAV8501 | Stripe rust | Add/Dom | 7,75 |  |  |
| IACX11417 | Stripe rust | Add/Dom | 4,97 |  |  |
| IACX6178 | Stripe rust | Add/Dom | 8,51 |  |  |
| Jagger_c5046_63 | Stripe rust |  |  | Add | 3,19 |
| Jagger_c5046_63 | Stripe rust | Dom | 2,74 |  |  |
| Jagger_c7212_85 | Stripe rust | Dom | 5,54 |  |  |
| Kukri_c12648_434 | Stripe rust | Add/Dom | 8,26 |  |  |
| Kukri_c1468_1609 | Stripe rust |  |  | Add | 1,28 |
| Kukri_c22599_114 | Stripe rust | Add/Dom | 7,28 |  |  |
| Kukri_c23195_266 | Stripe rust | Add/Dom | 7,86 |  |  |
| Kukri_c31776_1621 | Stripe rust | Add/Dom | 7,87 |  |  |
| Ra_c26532_894 | Stripe rust | Add/Dom | 7,92 |  |  |
| Ra_c6266_136 | Stripe rust | Dom | 5,54 |  |  |
| Ra_c6266_140 | Stripe rust | Dom | 5,54 |  |  |
| RAC875_c1226_652 | Stripe rust |  |  | Add | 0,19 |
| RAC875_c1226_652 | Stripe rust | Dom | 8,01 |  |  |
| RAC875_c26214_505 | Stripe rust | Add/Dom | 5,74 |  |  |
| RAC875_c47161_100 | Stripe rust | Add | 6,17 |  |  |
| RAC875_c5577_1682 | Stripe rust | Add/Dom | 10,92 |  |  |
| RAC875_c829_1143 | Stripe rust | Add/Dom | 5,58 |  |  |
| RAC875_c829_1215 | Stripe rust | Add/Dom | 4,99 |  |  |
| RAC875_c8721_212 | Stripe rust |  |  | Add | 3,30 |
| RAC875_c8721_212 | Stripe rust | Dom | 2,68 |  |  |
| RAC875_rep_c107961_348 | Stripe rust | Dom | 3,34 |  |  |
| RAC875_rep_c113106_93 | Stripe rust | Add/Dom | 7,49 |  |  |
| RFL_Contig174_406 | Stripe rust | Add | 12,03 |  |  |
| RFL_Contig5262_1500 | Stripe rust |  |  | Add | 1,28 |
| TA002254-0660 | Stripe rust | Add/Dom | 15,83 |  |  |
| TA003766-0683 | Stripe rust | Add/Dom | 7,04 |  |  |
| TA005377.1076 | Stripe rust |  |  | Add | 0,94 |
| Tdurum_contig11802_864 | Stripe rust | Add/Dom | 7,63 |  |  |
| Tdurum_contig29607_413 | Stripe rust |  |  | Add | 3,09 |
| Tdurum_contig29607_413 | Stripe rust | Dom | 2,31 |  |  |
| Tdurum_contig29983_490 | Stripe rust | Add/Dom | 7,98 |  |  |
| Tdurum_contig63196_123 | Stripe rust | Add/Dom | 8,32 |  |  |
| tplb0041b11_529 | Stripe rust | Add/Dom | 6,91 |  |  |
| wsnp_Ex_c19516_28481857 | Stripe rust | Add/Dom | 12,07 |  |  |
| wsnp_Ex_c2772_5130007 | Stripe rust | Add/Dom | 10,93 |  |  |
| wsnp_JD_c9251_10121369 | Stripe rust | Dom | 5,63 |  |  |
| wsnp_Ku_c23598_33524490 | Stripe rust | Dom | 9,25 |  |  |
| wsnp_Ku_rep_c113718_96236830 | Stripe rust |  |  | Add | 0,40 |
| wsnp_RFL_Contig1951_1127302 | Stripe rust | Add/Dom | 5,14 |  |  |
| wsnp_RFL_Contig4456_5258284 | Stripe rust |  |  | Add | 2,82 |
| wsnp_RFL_Contig4456_5258284 | Stripe rust | Dom | 2,72 |  |  |
